# Design and Synthesis of Poly(2,2′-Bipyridyl) Ligands for Induction of Cell Death in Cancer Cells: Control of Anticancer Activity by Complexation/Decomplexation with Biorelevant Metal Cations

**DOI:** 10.1021/acs.inorgchem.3c01738

**Published:** 2023-08-29

**Authors:** Chandrasekar Balachandran, Masumi Hirose, Tomohiro Tanaka, Jun Jie Zhu, Kenta Yokoi, Yosuke Hisamatsu, Yasuyuki Yamada, Shin Aoki

**Affiliations:** † Faculty of Pharmaceutical Sciences, Tokyo University of Science, 2641 Yamazaki, Noda 278-8510, Japan; ‡ Research Institute for Biomedical Sciences, Tokyo University of Science, 2641 Yamazaki, Noda, Chiba 278-8510, Japan; § Graduate School of Pharmaceutical Sciences, Nagoya City University, 3-1 Tanabe-dori, Nagoya, Aichi 467-8603, Japan; ∥ Department of Chemistry, Graduate School of Science, Nagoya University, Furo-cho, Chikusa-ku, Nagoya 464-8602, Japan; ⊥ Research Center for Materials Science, Nagoya University, Furo-cho, Chikusa-ku, Nagoya 464-8602, Japan; # Research Institute for Science and Technology, Tokyo University of Science, 2641 Yamazaki, Noda, Chiba 278-8510, Japan

## Abstract

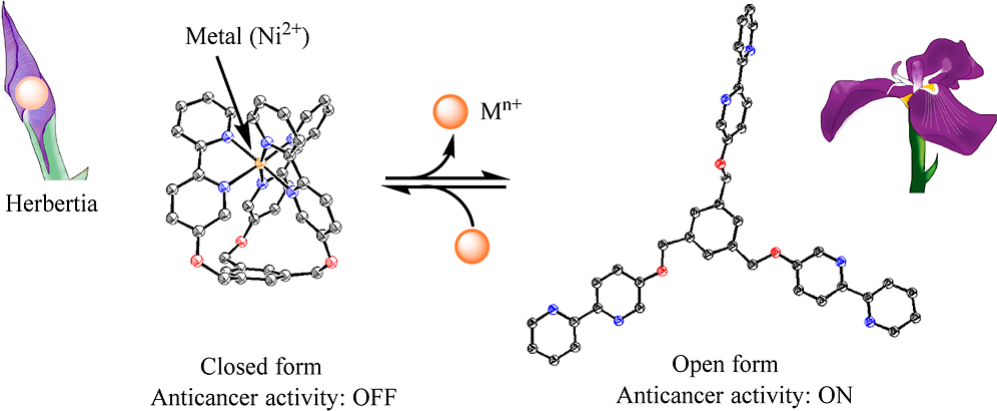

Chelation therapy is a medical procedure for removing toxic metals from human organs and tissues and for the treatment of diseases by using metal-chelating agents. For example, iron chelation therapy is designed not only for the treatment of metal poisoning but also for some diseases that are induced by iron overload, cancer chemotherapy, and related diseases. However, the use of such metal chelators needs to be generally carried out very carefully, because of the side effects possibly due to the non-specific complexation with intracellular metal cations. Herein, we report on the preparation and characterization of some new poly(bpy) ligands (bpy: 2,2′-bipyridyl) that contain one–three bpy ligand moieties and their anticancer activity against Jurkat, MOLT-4, U937, HeLa S3, and A549 cell lines. The results of MTT assays revealed that the tris(bpy) and bis(bpy) ligands exhibit potent activity for inducing the cell death in cancer cells. Mechanistic studies suggest that the main pathway responsible for the cell death by these poly(bpy) ligands is apoptotic cell death. It was also found that the anticancer activity of the poly(bpy) ligands could be controlled by the complexation (anticancer activity is turned OFF) and decomplexation (anticancer activity is turned ON) with biorelevant metal cations. In this paper, these results will be described.

## Abstract 2

New Herbertia-like poly(bpy) ligands are designed and synthesized. The anticancer activity of the poly(bpy) ligands is turned OFF upon the complexation (closed form) with biorelevant metal ions and turned ON (open form) by the decomplexation.

## Introduction

Chelation therapy is a medical procedure for removing toxic metals from human organs and tissues by using metal-chelating agents.^1,2^ For example, iron chelation therapy was designed to treat metal poisoning and iron overload diseases, and the interest in some of the iron chelators has shifted to their use in the treatment of cancer,^3−11^ neurodegeneration disease,^12^ and related diseases.^13^ Iron is one of the more important elements in all living organisms and plays important roles in biochemical cellular processes such as energy metabolism and DNA synthesis. Importantly, cancer cells require more iron ions than normal cells to mediate their rapid DNA synthesis and growth and to suppress the proliferation of cancer stem cells, which are important therapeutic targets.^14^

**Scheme 1:**
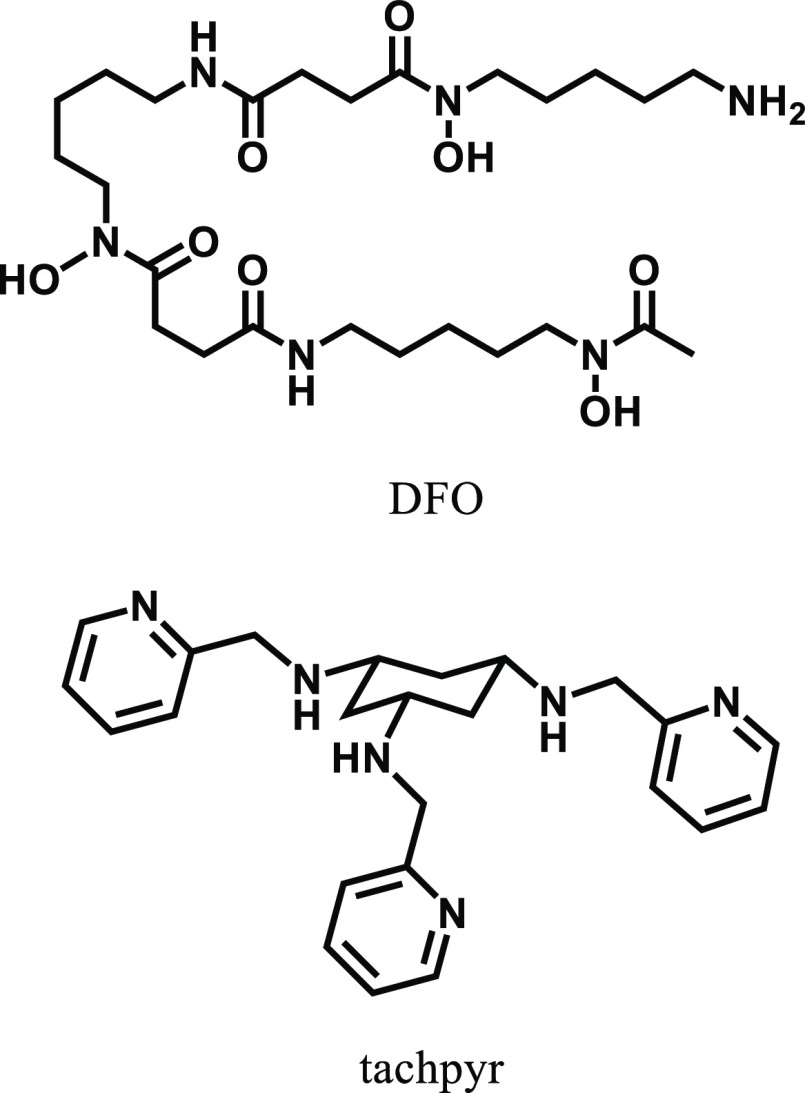
Structures of Representative Chelators for Chelation Therapy

For the above reasons, the development of novel chelators for intracellular metal cations such as Fe^2+^, Cu^2+^, and Zn^2+^ represents a potentially promising anticancer strategy for addressing these diseases. For example, desferrioxamine (DFO), a first line agent of the treatment of an iron overload, was utilized in clinical trials for the treatment of hepatocellular carcinoma (Scheme 1).^15^ It has also been reported that copper chelators induce the cell death in colon cancer cells via the generation of reactive oxygen species.^16−19^ Tripodal ligands such as *N*,*N′*,*N*″-tris(2-pyridylmethyl)-*cis*,*cis*-1,3,5-triaminocyclohexane (tachpyr)^20−23^ provide additional donor sites and affect the steric environment around the metal center allowing more efficient metal chelating, resulting in the activation of intracellular cell death mechanisms. However, care needs to be exercised in the use of metal chelators, because of the side effects possibly due to the nonspecific complexation with intracellular metal cations.

**Scheme 2:**
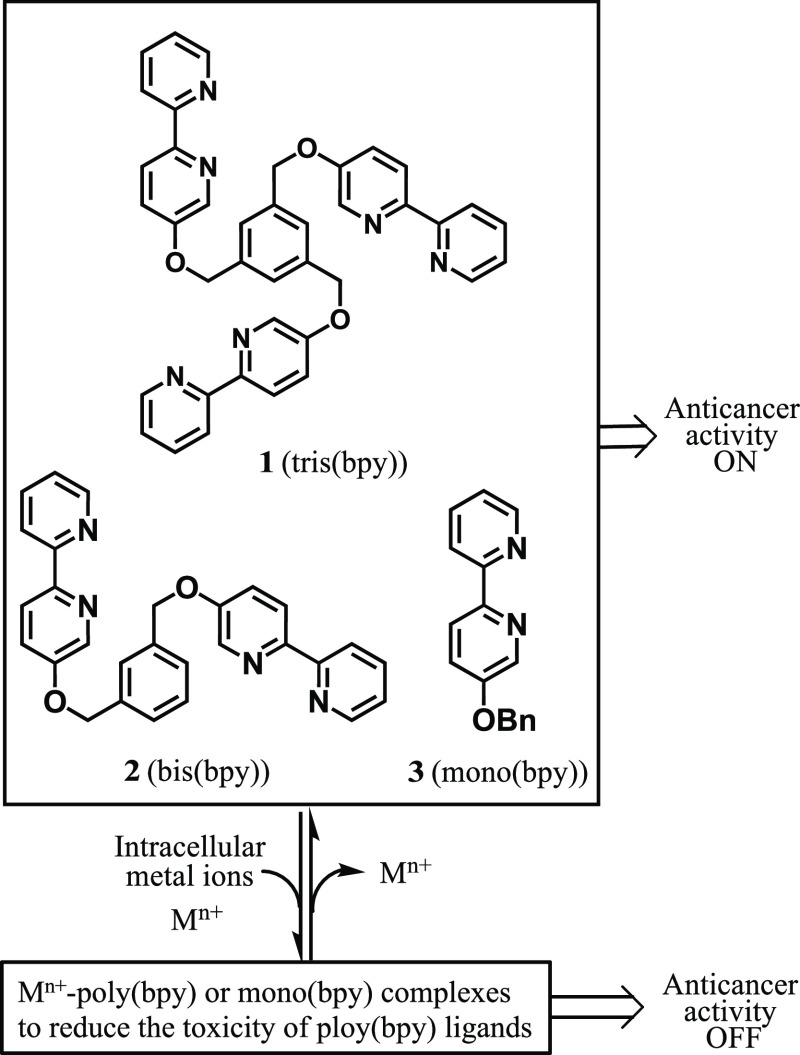
Structures of Poly(2,2′-Bipyridyl) Ligands and the Proposed Scheme for the Control of This Anticancer Activity by Complexation with Intracellular Metals

This background has prompted us to focus on the design and synthesis of poly(bpy) ligands (bpy: 2.2′-bipyridyl) as anticancer agents, because bpy is the most widely used lipophilic metal chelators for many purposes.^24^ In this work, we report on the synthesis of a tris(bpy) ligand **1** and an assessment of its cytotoxicity against cancer cell lines (Scheme 2). The bis(bpy) ligands **2** and the mono(bpy) ligand **3** were also synthesized for comparison. The results of the evaluation of anticancer activity of these molecules indicate that **1** exhibited the most potent cytotoxicity against blood cancer cell lines (Jurkat, MOLT-4 and U937 cells), and that **1** is more potent against cancer cell lines than against normal cells. The X-ray crystal structures of metal-free **1** and its Ni^2+^ complex and mechanistic aspects of the induction of cancer cell death are reported. However, neurotoxicity of 2,2′-bpy as well as 2,3′- and 4,4′-bpy derivatives has been reported.^25^

**Scheme 3:**
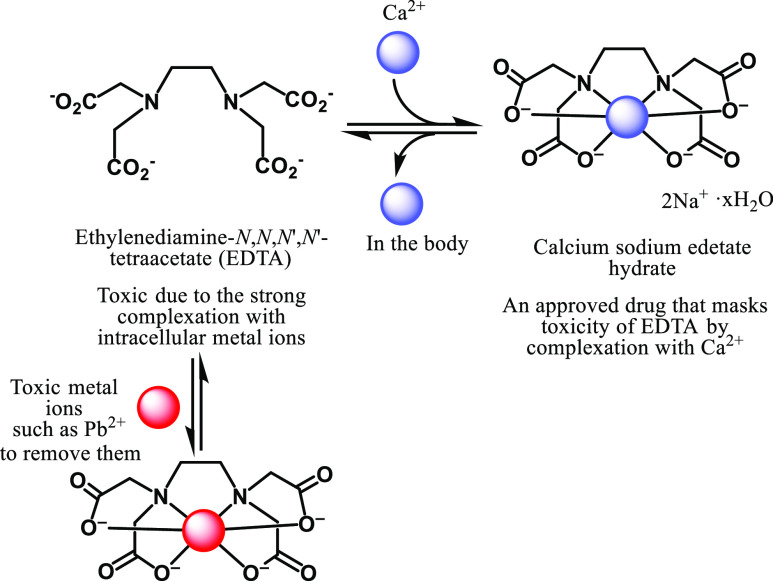
Proposed Scheme of Calcium Sodium Edetate Hydrate (CaNa-EDTA)

The chelation therapy using potent metal chelators may suffer from their toxicity due to the strong binding property with intracellular metal ions.^26^ For example, ethylenediaminetetraacetic acid (EDTA) has been known as one of very strong metal chelators and used for the reduction of the toxicity of heavy metals (Scheme 3). EDTA itself is toxic and has been approved as a complex with calcium and sodium (calcium sodium edetate hydrate) to mask its toxicity.^27^ In the body, metal-free EDTA is released and toxic metal ions such as lead (Pb^2+^) are trapped and excreted from the body.

This information has prompted us to use metal complexes of tris(bpy) and bis(bpy) ligands for the time-dependent release of their metal-free forms. Namely, metal complexes of the poly(bpy) ligands with typical intracellular metal cations such as Fe^2+^, Co^2+^, Ni^2+^, Cu^2+^, and Zn^2+^ were prepared in the second half of this manuscript. The stability of these metal complexes and time-dependent change in their anticancer activity due to decomplexation are examined. In addition, these anticancer properties were compared with cisplatin, actinomycin D, and one of the most potent metal chelators, *O*,*O*′-bis(2-aminophenyl)ethyleneglycol-*N*,*N*,*N*′,*N*′-tetraacetic acid (BAPTA), and its acetoxymethyl ester (BAPTA-AM).

## Results and Discussion

### Synthesis of Poly(bpy) Ligands

**Scheme 4:**
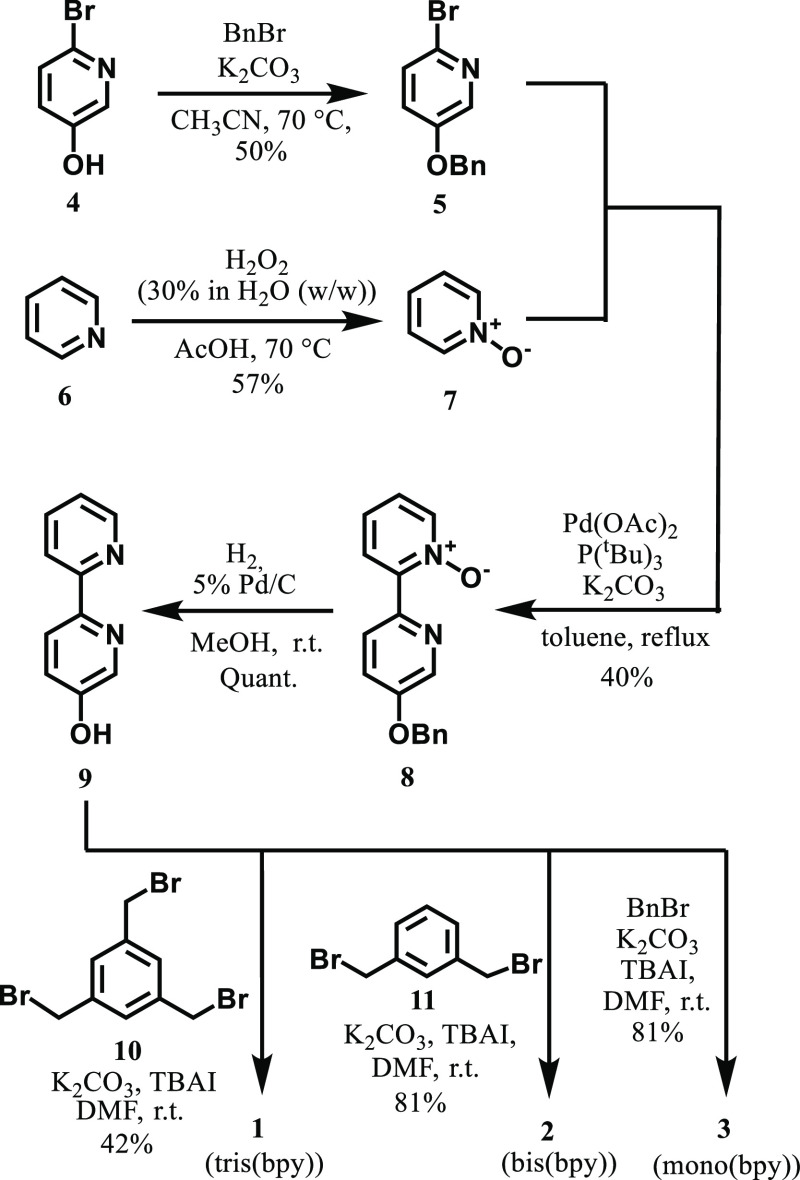
Synthesis of poly(bpy) and mono(bpy) Ligands **1**–**3**

The synthesis of poly(bpy) ligands **1**–**3** was carried out as shown in Scheme 4. In the first step, 2-bromo-5-hydroxypyridine **4** was reacted with benzyl bromide to obtain **5**.^28^ A coupling reaction of **5** with pyridine *N*-oxide **7** prepared from pyridine **6**(29) according to the previously reported methodology gave **8**,^30^ which was subjected to the reduction to give **9**. Next, **9** was reacted with 1,3,5-tris(bromomethyl)benzene **10** to give the tris(bpy) ligand **1**. As references, the bis(bpy) ligand **2** and the mono(bpy) ligand **3** were prepared by reactions with 1,3-bis(bromomethyl)benzene **11** and benzyl bromide, respectively, in a similar manner to that of **1**. The ligands **1**–**3** are soluble in DMSO and their stock solutions in DMSO were prepared and used in the following biological experiments.

### Cytotoxicity of Tris(bpy), Bis(bpy), and Mono(bpy) Ligands against Different Cell Lines, as Evaluated by MTT Assays

**Figure 1:**
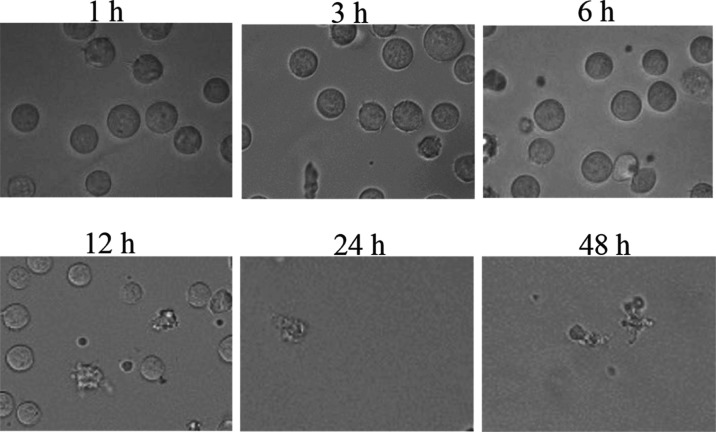
Time-dependent morphological change in Jurkat cells after incubation with **1** (1 μM) for 1–48 h (magnification: ×120).

First, morphological change in Jurkat cells (human T-lymphocyte leukemia) was observed in microscopy after the incubation with **1** for 1–48 h to see whether **1** would induce cell death in Jurkat cells or not. As shown in Figure 1, Jurkat cells presented apoptotic like body formation, indicative of the typical morphology associated with apoptosis.^31−33^

**Figure 2:**
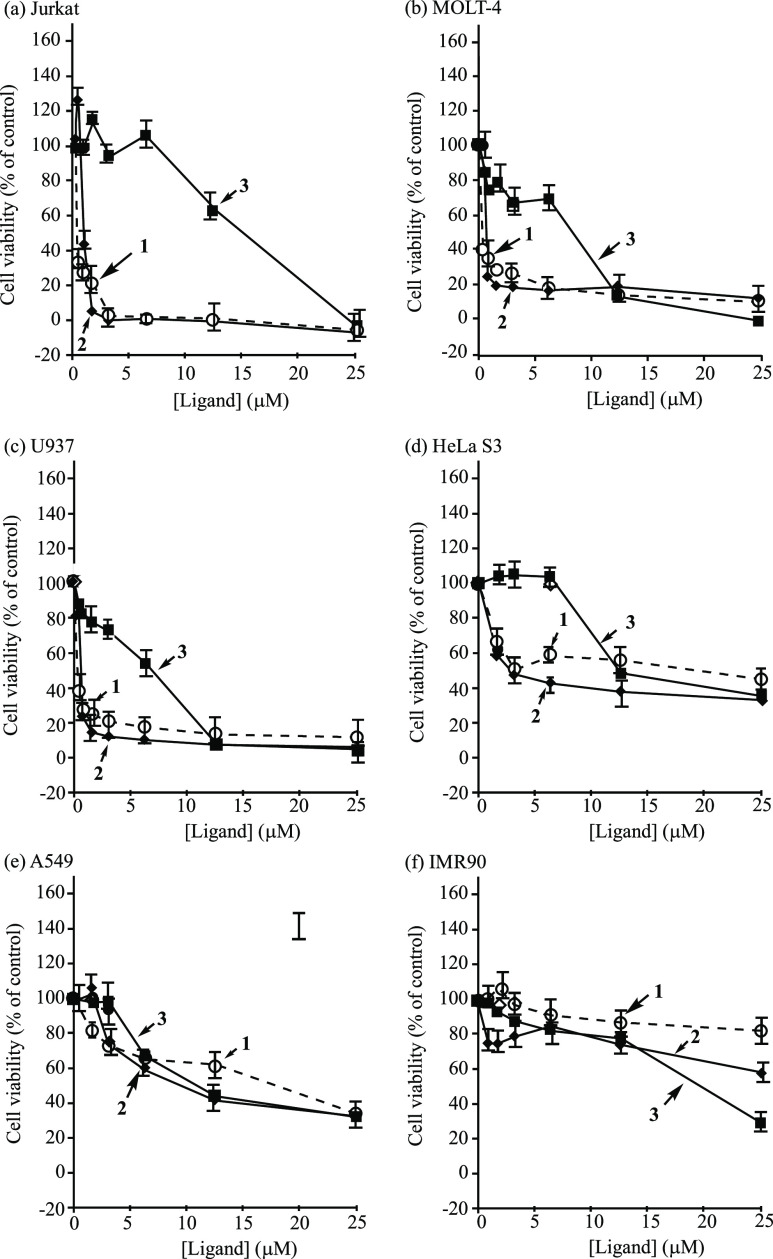
Results of MTT assay of (a) Jurkat cells, (b) MOLT-4 cells, (c) U937 cells, (d) HeLa S3 cells, (e) A549 cells, and (f) IMR-90 cells treated with **1** (dashed curve with open circles), **2** (plain curve with filled diamonds), and **3** (plain curve with filled squares) for 24 h.

Second, we conducted the quantitative evaluation of the cytotoxicity of **1**, **2**, and **3** against various cancer cell lines by an MTT (3-(4,5-dimethylthiazol-2-yl)-2,5-diphenyltetrazolium bromide) assay. Jurkat, MOLT-4 (these two cell lines are human T-lymphocyte leukemia cells), and U937 (human lymphoma) cells (1.5 × 10^4^ cells/well) were incubated in RPMI 1640 medium containing 10% FBS (fetal bovine serum) containing **1**, **2**, and **3** (0–25 μM) at 37 °C for 24 h, and then treated with the MTT reagent. A549 (human alveolar adenocarcinoma) and IMR-90 (normal human Caucasian fetal lung fibroblast as model of normal cells) cells (1.5 × 10^4^ cells/well) were incubated in DMEM (low glucose) with 10% FBS containing **1**, **2**, and **3** (0–25 μM), HeLa S3 (human epithelial carcinoma cells) cells (1.5 × 10^4^ cells/well) were incubated in MEM with 10% FBS containing these ligands (0–25 μM) for 24 h at 37 °C and then treated with the MTT reagent. The results of the MTT assays against Jurkat, MOLT-4, U937, HeLa S3, A549, and IMR-90 cells are summarized in Figure 2 and Table 1. The results of MTT assays suggested that **1** and **2** exhibited more potent cytotoxicity against Jurkat, MOLT-4, U937, and HeLa S3 cells than **3**, and that **1**–**3** are more potent against these cancer cell lines than against a model of normal cells IMR-90.

**Table 1:** EC_50_ Values (μM) of **1**, **2**, **3**, Cisplatin, Actinomycin D, BAPTA, and BAPTA-AM against Jurkat, MOLT-4, U937, A549, HeLa S3, and IMR-90 Cells Obtained by MTT Assays after Incubation at 37 °C for 24 or 48 h

compounds	Jurkat	MOLT-4	U937	HeLa S3	A549	IMR-90
**1**	0.14 ± 0.13^a^ ((6.4 ± 0.21) x 10^–2^_)_^b^	0.21 ± 0.36^a^(<0.1)^b^	0.32 ± 0.22^a^	16 ± 0.33^a^ (2.8 ± 1.6)^b^	15 ± 0.79^a^	>100^a^
2	0.77 ± 0.56^a^	0.64 ± 0.55^a^	0.59 ± 0.33^a^	15 ± 1.34^a^	9.5 ± 1.2^a^	>100^a^
**3**	13 ± 0.34^a^	8.7 ± 0.89^a^	5.6 ± 0.77^a^	15 ± 0.37^a^	11 ± 0.55^a^	75 ± 0.1^a^
cisplatin	33 ± 0.2^a^	14 ± 0.3^a^	24 ± 0.9^a^	29 ± 1.2^a^	>100^a^	>100^a^
actinomycin D	(5.0 ± 0.11) x 10^–3^^a^	(3.0 ± 0.16) x 10^–3^^a^	(5.1 ± 0.6) x 10^–2^^a^	>10^a^	64 ± 1.1^a^	53 ± 2.6^a^
BAPTA	>50^a^	>50^a^	>50^a^	>50^a^	>50^a^	>50^a^
BAPTA-AM	8.6 ± 0.3^a^	13.2 ± 0.2^a^	7.1 ± 0.4^a^	>50^a^	>50^a^	>50^a^

a Cells were treated with drugs for 24 h.

b Cells were treated with drugs for 48 h.

**Scheme 5:**
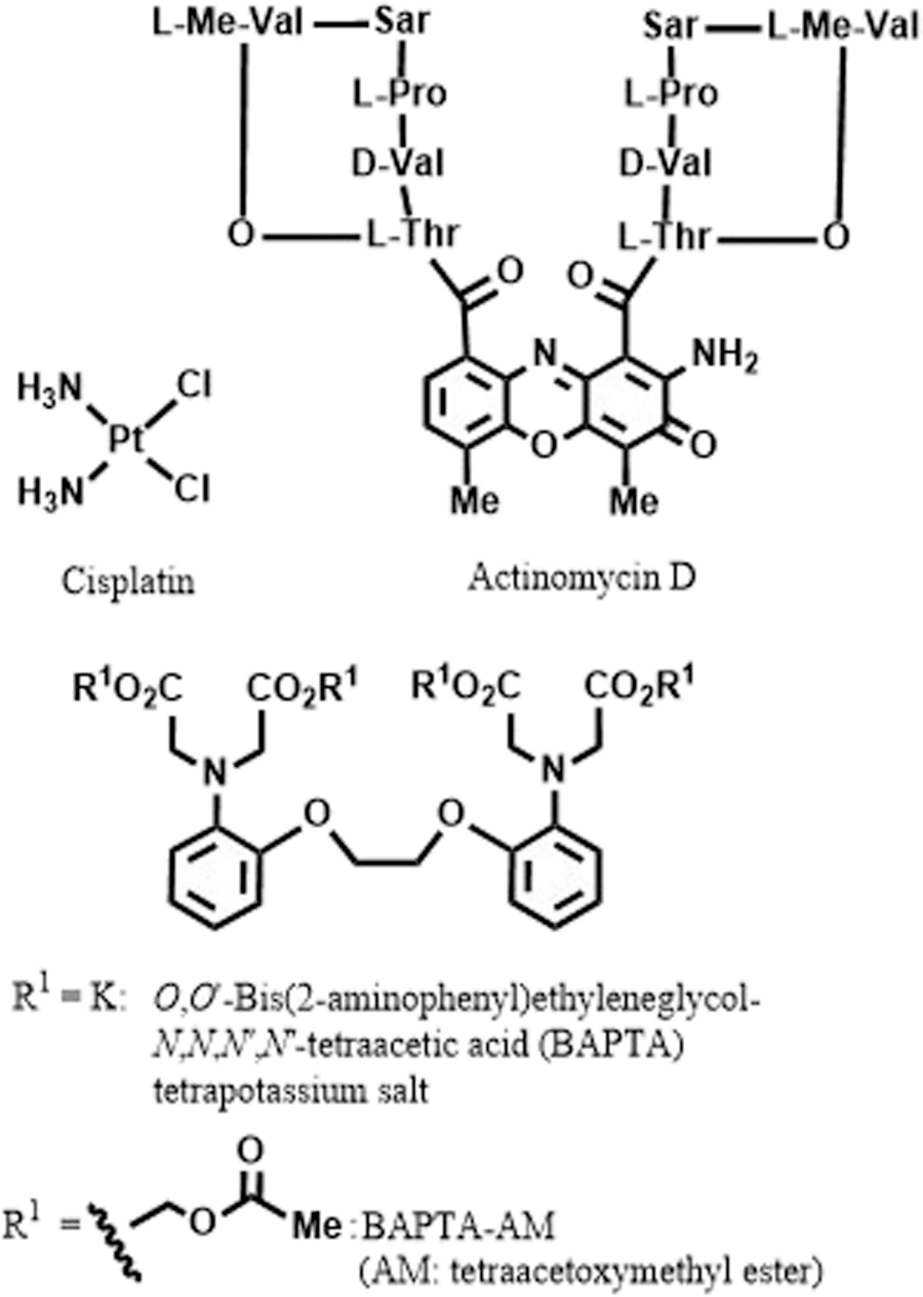
Structures of Cisplatin, Actinomycin D, BAPTA (K^+^ Salt), and BAPTA-AM

For comparison, the EC_50_ values of cisplatin, actinomycin D, BAPTA (known as a Ca^2+^ chelator that assists apoptosis),^34−37^ and its acetoxymethyl (AM) ester (BAPTA-AM) were also determined (Scheme 5) as summarized in Table 1 and Figure S1 in the Supporting Information, which indicates that actinomycin D was potent against these cancer cell lines. These data prompted us to mainly use **1** in subsequent experiments for the purpose of controlling anticancer activity by complexation and decomplexation with biorelevant metal ions.

### Mechanistic Study of the Cell Death in Jurkat Cells Induced by Poly(bpy) Ligands

**Figure 3:**
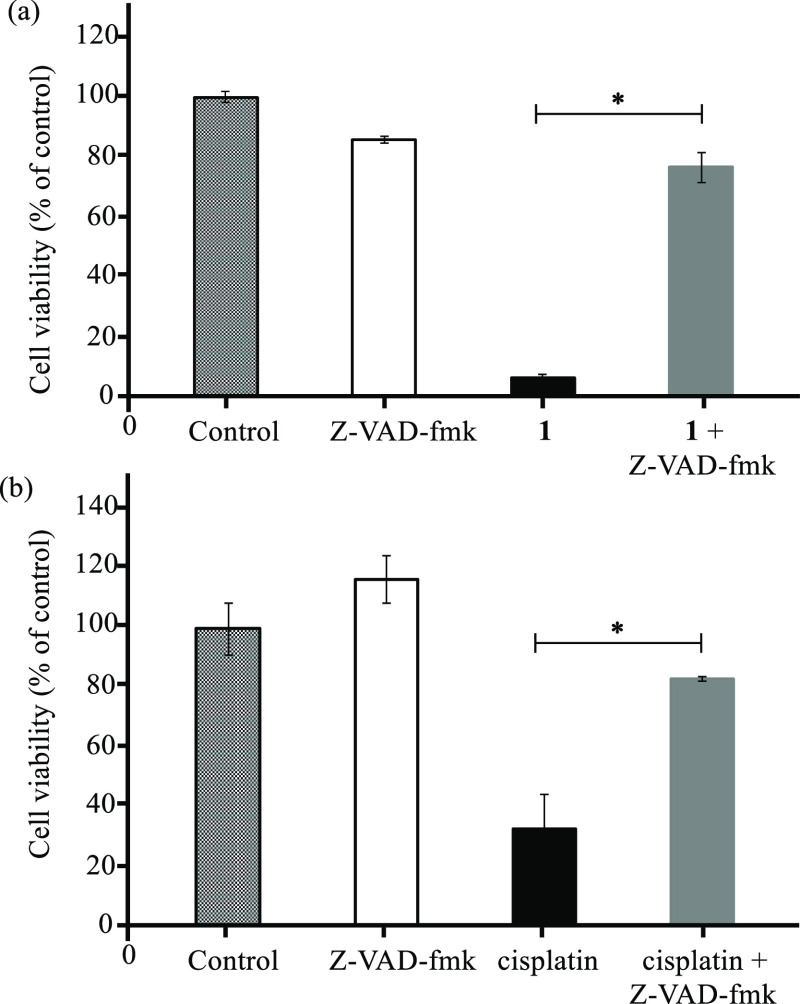
Effect of Z-VAD-fmk on the cell death in Jurkat cells induced by **1**. Jurkat cells were incubated in RPMI in the presence of Z-VAD-fmk (15 μM) for 3 h and then treated with (a) **1** (1 μM) and (b) cisplatin (50 μM) for 24 h. Asterisks indicate significant difference with *(*P* ≤ 0.05).

The results of MTT assays presented in Table 1 indicate that **1** exhibits potent anticancer activity against Jurkat cells than against other cancer cells. We, therefore, selected Jurkat cells to study the cell death mechanism in detail, in this work. Programmed cell death (PCD) is generally classified into several types such as apoptosis, necroptosis, paraptosis, autophagic cell death, and so on.^30−33^ We hypothesized that **1** and **2** function as inducers of apoptosis, which is a type of PCD in cancer cells, based on the morphological change of Jurkat cells observed in Figure 1 and mechanism of chelation therapy proposed in the previous studies.^11−19^ It is known that the caspases family are cysteine proteases that play important roles in apoptosis and necroptosis.^38^ Therefore, the effect of Z-VAD-fmk, which is known as one of general caspase inhibitors, on the cell death induced by **1** and cisplatin was examined. As shown in Figure 3, cell viability of Jurkat cells was considerably restored by the preincubation of Jurkat cells with Z-VAD-fmk for 3 h prior to the treatment with **1** (1 μM) or cisplatin (50 μM) for 24 h.

**Figure 4:**
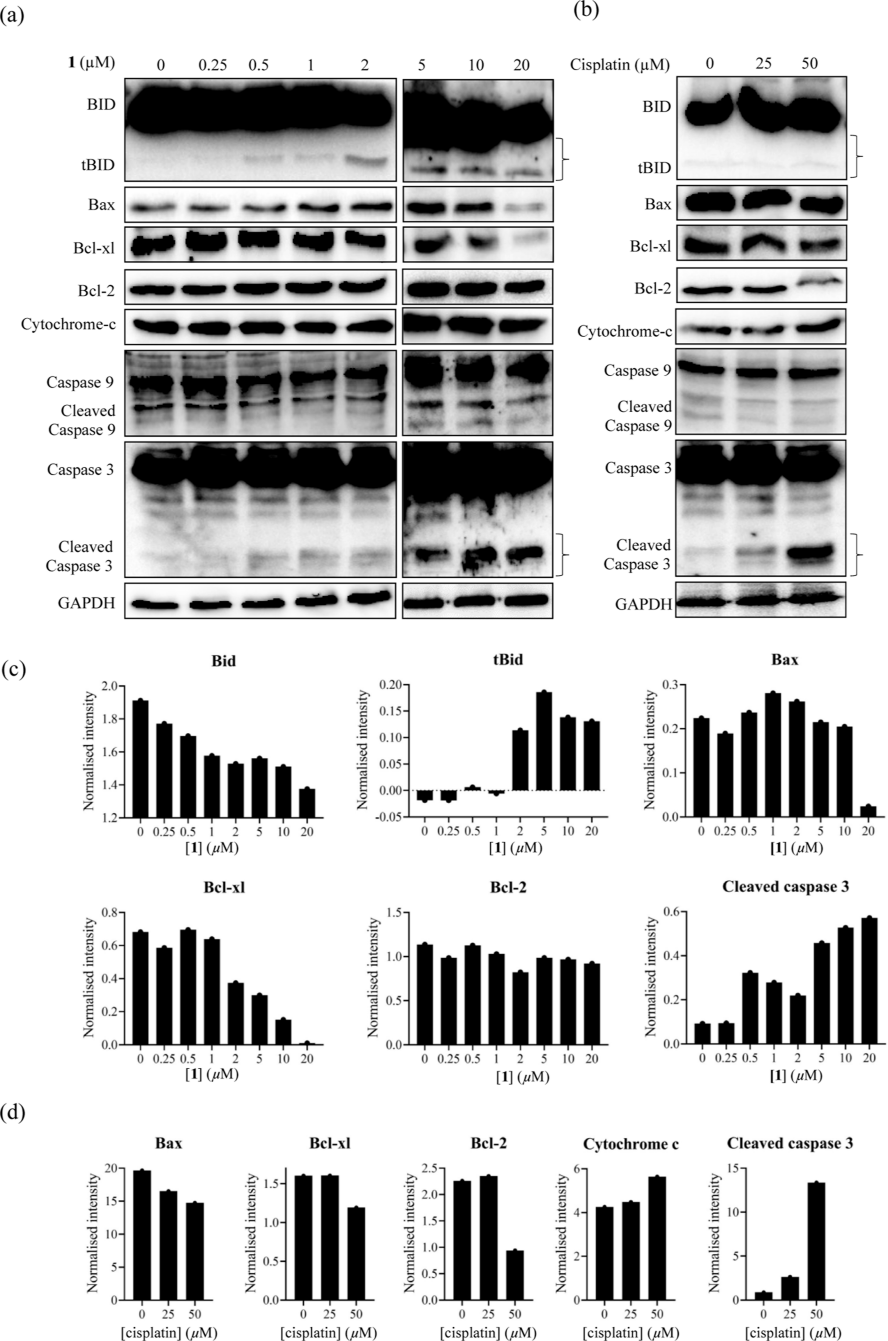
Effects of **1** and cisplatin on the expression (and cleavage) of BID, Bax, Bcl-2, Bcl-xl, cytochrome *c*, caspase-9, and caspase-3. Jurkat cells were treated with **1** (0, 0.25, 0.5, 1, 2, 5, 10, 20 μM) (a,c) and cisplatin (0, 25, 50 μM) (a,c) for 24 h. Expression of BID, Bax, Bcl-2, Bcl-xl, cytochrome-c, caspase-9, and caspase-3 were analyzed by a Western blot analysis. Expression of GAPDH was set as a protein loading control.

Next, we performed a Western blot of apoptosis-related proteins such as BID, Bax, Bcl-xl, Bcl-2, cytochrome *c*, caspase 3, and caspase 9 in Jurkat cells after the treatment with **1** and cisplatin to analyze the cell death mechanism. Jurkat cells were incubated with **1** and cisplatin for 24 h at concentrations ([**1**] = 0.25–20 μM and [cisplatin] = 25–50 μM) close to the EC_50_. The results of Western blot indicated the cleavage of caspase-3 by both compounds (Figure 4). The results for **1** indicated the activation of tBID and the suppression of Bax and Bcl-xl, while cisplatin decreased the Bax, Bcl-xl, and Bcl-2, and increased cytochrome *c* as well as the cleavage of caspase-3.

**Figure 5:**
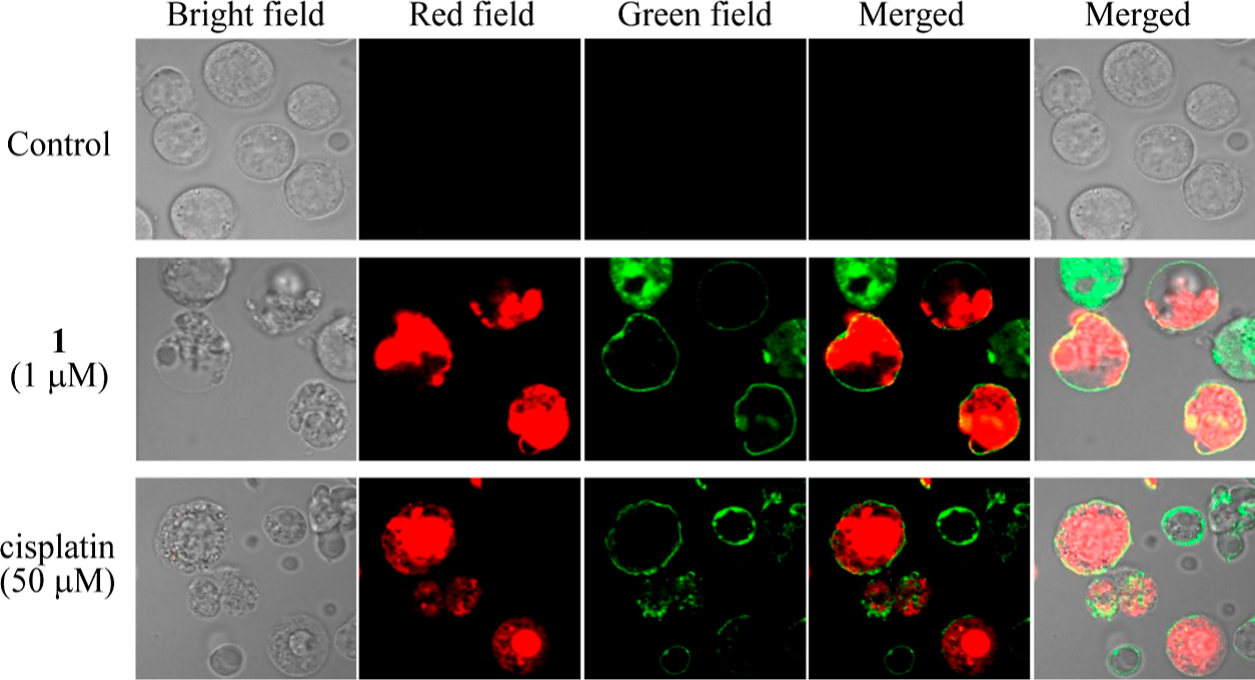
Fluorescence microscopy images of Jurkat cells after the treatment with **1** (1 μM) and cisplatin for 24 h, and Annexin V-FITC/PI. The samples were analyzed for green fluorescence (FITC) and red fluorescence (PI).

We also carried out Annexin V-FITC/propidium iodide (PI) staining experiments of Jurkat cells after the treatment with **1** by confocal microscopy, because a combination of PI and Annexin V-FITC is widely used to discriminate apoptosis from necrosis in cells.^39^ In the present study, Jurkat cells (1.0 × 10^5^ cells/tube) were incubated at 37 °C for 24 h in the presence of **1** (1 μM) and cisplatin (50 μM) and then stained with Annexin V-FITC (λ_ex_ = 495 nm, λ_em_ = 520 nm). It is known that phosphatidylserine (PS) is translocated from the inner side of the plasma membrane to the outer layer (flip-flop) in apoptotic cells and that Annexin V-FITC is a phospholipid-binding protein with a high affinity for PS, which can be used as a probe for expressed PS that is expressed on the cell surface. As shown in Figure 5, a green emission from Annexin V-FITC on the cell membrane and red emission from PI in cytoplasm were observed.

**Figure 6:**
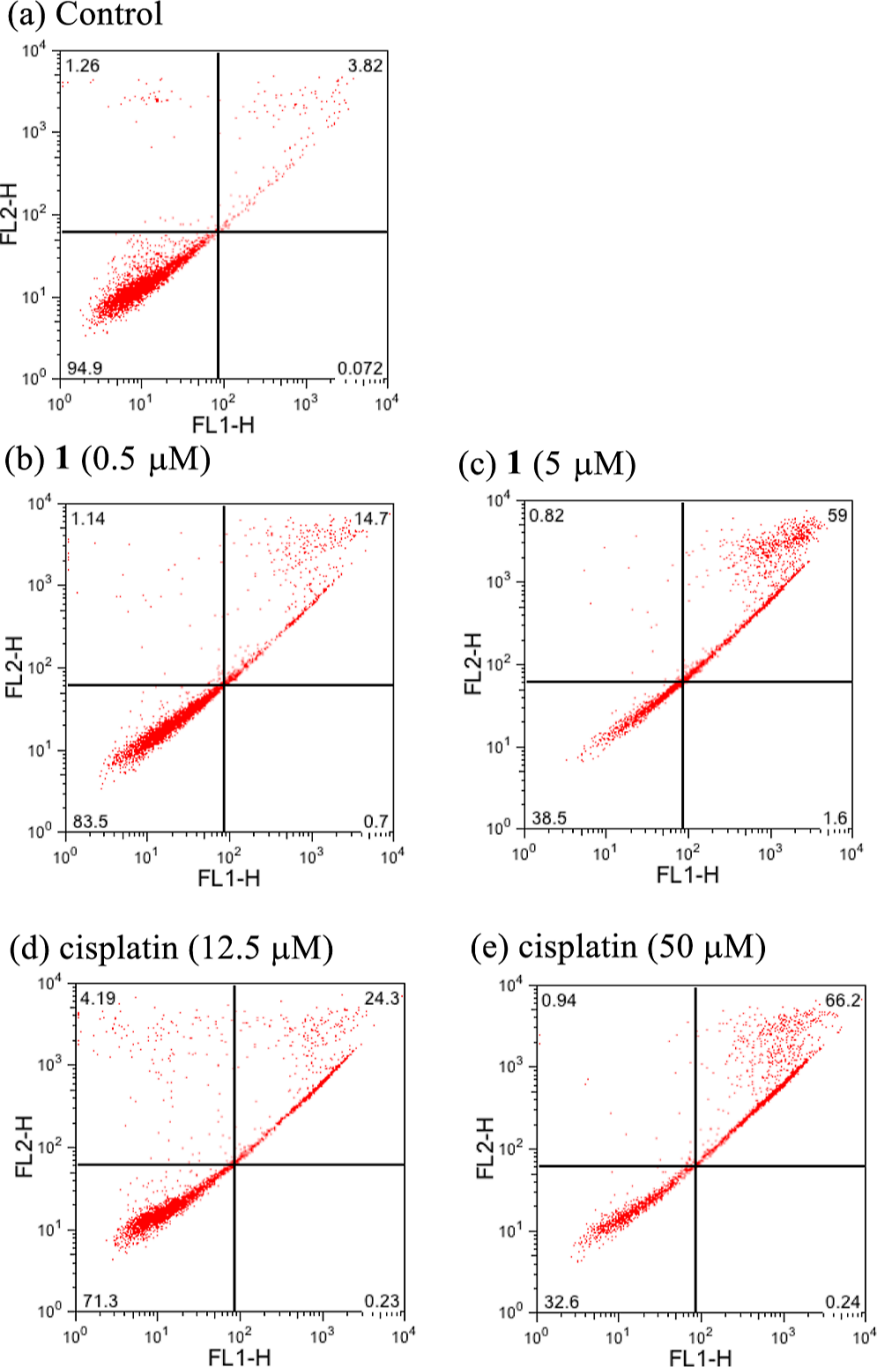
Jurkat cells were treated with no ligand (a) and with **1** (0.5–5 μM) (b,c) and cisplatin (12.5–50 μM) (d,e) and for 24 h, stained with Annexin V-FITC/PI, and observed by flow cytometry. The four areas in each graph show necrotic cells in the top left square (Annexin V-FITC^-^/PI^+^), late apoptotic cells in the top right square (Annexin V-FITC^+^/PI^+^), alive cells in the bottom left square (Annexin V-FITC^–^/PI^–^), and apoptotic cells in the bottom right square (Annexin V-FITC^+^/PI^–^).

**Figure 7:**
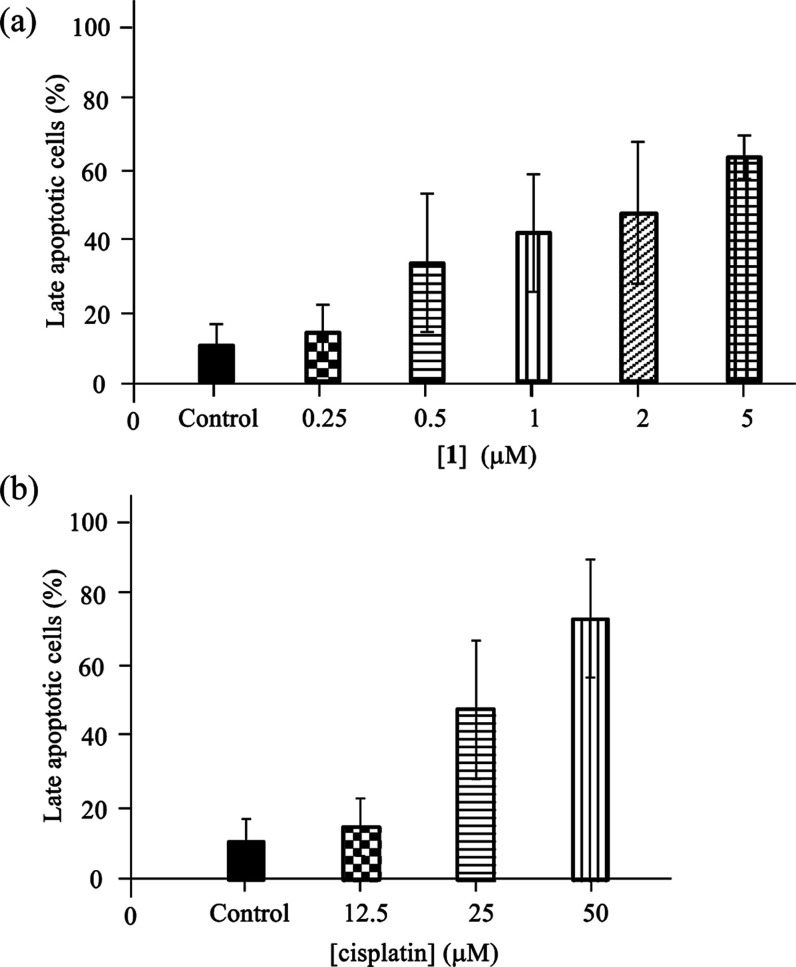
Ratio (%) of the late apoptotic/necrotic stage of Jurkat cells after the treatment with **1** (0.25–5 μM) for 24 h (a) and cisplatin (12.5–50 μM) for 24 h (b) (*n* = 3).

The cell death in Jurkat cells induced by **1** and cisplatin (after the treatment for 24 h) was characterized by the flow cytometry of Jurkat cells after co-staining with Annexin V-FITC and PI to characterize the early and late apoptosis or necrosis. Each of the four areas in Figure 6 indicates Annexin V-FITC negative and PI signal positive (= necrotic cells) (top left square), Annexin V-FITC positive and PI positive (= late apoptosis) (top right square), Annexin V-FITC negative and PI negative (= alive cells) (bottom left square); and Annexin V-FITC positive and PI signal negative (= early apoptosis cells) (bottom right square), respectively. As summarized in Figure 7, the percentage of late apoptotic cells of the cells that had been treated with cisplatin increased from 15 to 75% at [cisplatin] = 12.5 to 50 μM and the percentage of late apoptotic cells of cells treated with **1** increased from 14 to 65% at [**1**] = 0.25 to 5 μM. The collective data based on Figures 1, and 3–7 strongly suggest that **1** and cisplatin induce apoptosis and **1** is much potent than cisplatin.

### Relationship between Cytotoxicity of Metal Complexes of **1** and Their Kinetic Stability (Reactivation of **1** by the Decomplexation of Its Metal Complexes)

As described in the Introduction of the manuscript, it has been reported that 2,2′-bpy ligands possess anticancer activity,^26^ while their toxicity against normal tissues has also been described. Prior to the evaluation of suppression of anticancer activity of **1**–**3** against cancer cells by the complexation with metal ions, we measured the apparent stability constants (log *K*_s_) and the dissociation constants (*K*_d_) of **1** with biorelevant metal ions such as Zn^2+^, Ni^2+^, Co^2+^, Cu^2+^, and Fe^2+^. Namely, it is assumed that the more metal complexes are stable [the bigger the apparent stability constants (log *K*_s_) are, the smaller the dissociation constants (*K*_d_) are], the more anticancer activity **1** would be masked.

The UV/vis absorption titration experiments of **1** (30 μM) with Zn^2+^, Ni^2+^, Co^2+^, Cu^2+^, Fe^2+^, and A1^3+^ were conducted as shown Figure S2 in the Supporting Information, in which the 1:1 complexation of **1** with Zn^2+^, Ni^2+^, and Cu^2+^ was strongly suggested. From these titration curves, the apparent stability constants (log *K*_s_) of Zn^2+^-**1**, Ni^2+^-**1**, and Cu^2+^-**1** complexes were determined to be 6.56 ± 0.02, 6.21 ± 0.05, 7.29 ± 0.02, respectively, from which their dissociation constants *K*_d_ (= 1/*K*_s_) were calculated to be 0.28 ± 0.01, 0.61 ± 0.02, and 0.52 ± 0.02 μM, respectively (Table 2), indicating the almost identical stability of these three metal complexes.

**Table 2:** Apparent Stability Constants (log *K*_s_) of the Complexes of **1** (30 μM) with Metals in DMSO/30 mM HEPES [pH 7.4 with *I* = 0.1 (NaNO_3_)] (7/3) at 37 °C, Assuming 1:1 Complexation between **1** and Metals^a^

	Zn(NO_3_)_2_	NiSO_4_	Co(NO_3_)_2_	Cu(NO_3_)_2_	Fe(NO_3_)_2_	Al(NO_3_)_3_
log *K*	6.56 ± 0.02 (1:1 complex)	6.21 ± 0.01 (1:1 complex)	>6^b^ (1:2 complex)	7.29 ± 0.02 (1:1 complex)	>5.5^b^ (1:2 complex)	<1
*K*_d_ (μM)	0.28 ± 0.01	0.61 ± 0.02	n.d.^b^	0.05 ± 0.01	n.d.^b^	

a The UV/vis absorption titrations of **1** with metal ions were carried out by addition of 10 mM solutions of Zn(NO_3_)_2_, NiSO_4_, Co(NO_3_)_2_, Fe(NO_3_)_2_, and Al(NO_3_)_3_ in H_2_O and 10 mM solutions of Cu(NO_3_)_2_ in DMSO to solutions of **1** (30 μM) in DMSO/30 mM HEPES [pH 7.4 with *I* = 0.1 (NaNO_3_)] (7/3). The log *K*_s_ values were calculated based on the titration curves at given wavelengths for each metal ions, as indicated in the insets of Figure S2 of the Supporting Information.

b Only approximate *K*_d_ values for the 1:2 complexes of **1** with Co^2+^ and Fe^2+^ (by assuming 1:1 complexation) are shown and the *K*_d_ values are not determined (n.d.), because the unit for these 1:2 complexes is M^–2^, which is different from that for 1:1 complexes (M^–1^), which hampers their direct comparison (ref (40)).

On the other hand, the results of UV/vis absorption titrations of **1** with Co^2+^ and Fe^2+^ shown in Figure S2c,e in the Supporting Information suggest the 1:2 complexation of these metals with **1** to form Co^2+^-(**1**)_2_ and Fe^2+^-(**1**)_2_ complexes. Although simple comparison of the stability of metal complexes is difficult due to the different units for 1:1 complexation (M^–1^) and 1:2 complexation (M^–2^), it is likely that the thermodynamic stability of Co^2+^-(**1**)_2_ and Fe^2+^-(**1**)_2_ complexes are similar to that of Zn^2+^-**1**, Ni^2+^-**1**, and Cu^2+^-**1** complexes.^40^ Negligible change was observed in the titrations with Al^3+^ (Figure S2f in the Supporting Information), suggesting negligible complexation of **1** with Al^3+^.

**Figure 8:**
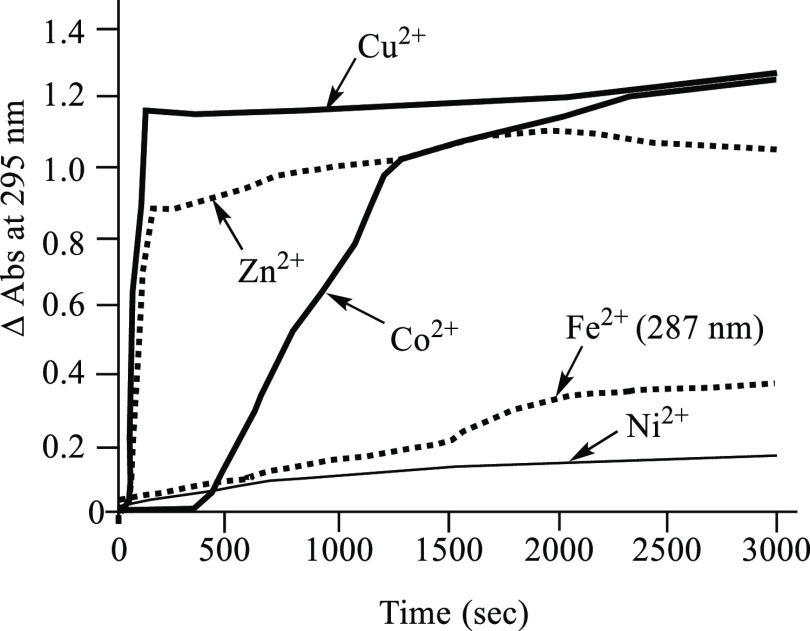
The change in UV/vis absorption spectra of the complexes of **1** (30 μM) with Cu^2+^, Co^2+^, Ni^2+^, and Zn^2+^ at 295 nm and complex of **1** with Fe^2+^ at 287 nm upon the addition of EDTA (450 μM) in DMSO/30 mM HEPES [(pH 7.4) with *I* = 0.1 (NaNO_3_)] (7/3) at 37 °C.

The kinetic stabilities of the aforementioned metal complexes were evaluated by the competitive methods.^41−45^ The change in UV/vis absorbance of the complexes of **1** (30 μM) with Cu^2+^, Co^2+^, Ni^2+^ and Zn^2+^at 295 nm was followed upon the addition of EDTA (450 μM) in DMSO/30 mM HEPES (pH 7.4 with *I* = 0.1 (NaNO_3_)) (7/3) at 37 °C. It is well known that EDTA forms very stable and well characterized complexes with Zn^2+^, Ni^2+^, Co^2+^, Cu^2+^, Fe^2+^, and Al^3+^, and their log *K*_s_ values were reported to be 16.3, 18.6, 16.0, 18.5, 14.0, and 16.1, respectively, in the literature.^46,47^ Indeed, a change in absorption spectra of the metal-**1** complexes was induced by the addition of EDTA, while negligible change was observed for Ni^2+^-**1** complex **12** (Figure 8). In addition, UV/vis absorption of the Zn^2+^ and Cu^2+^ complexes of **1** was restored to that of metal-free **1** more rapidly than those for Co^2+^ and Fe^2+^ complexes, suggesting that Zn^2+^, Cu^2+^, and Co^2+^ complexes of **1** are kinetically less stable than the corresponding Ni^2+^ and Fe^2+^ complexes.

**Figure 9:**
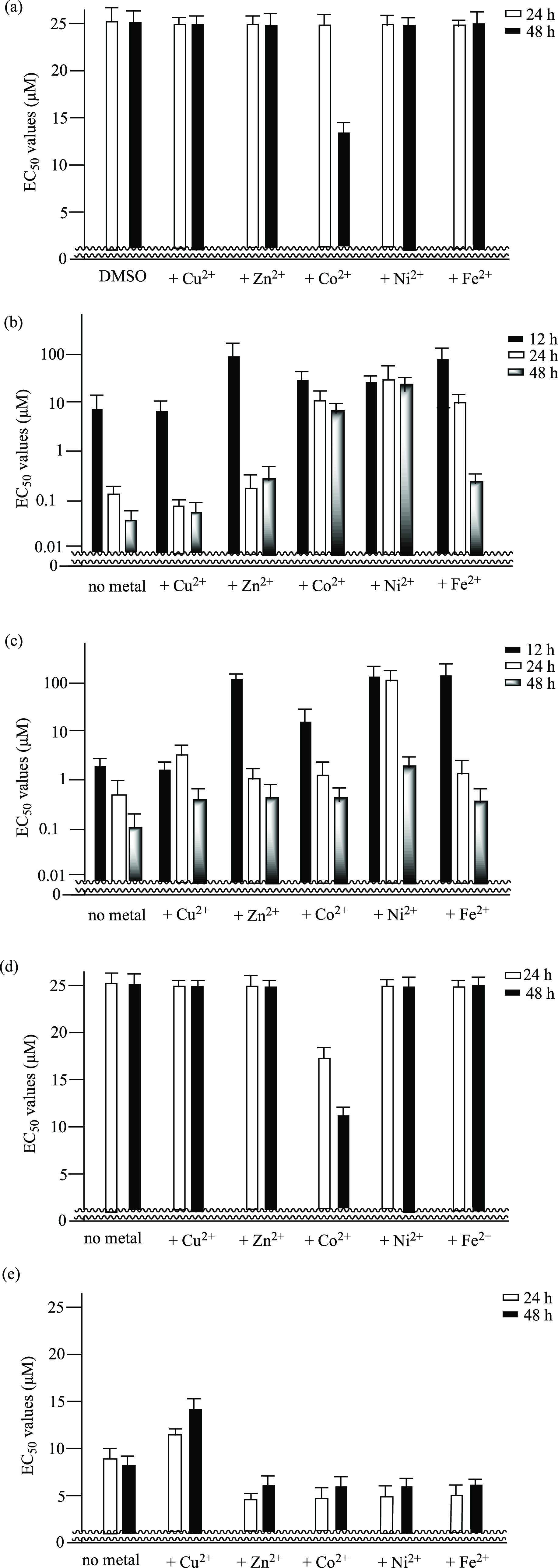
(a) The results of MTT assay of Jurkat cells treated with Cu(NO_3_)_2_, Zn(NO_3_)_2_, FeSO_4_, Co(NO_3_)_2_, and NiSO_4_, respectively, ([M^2+^] = 1.71–37.5 μM) for 24 h (closed bars) and 48 h (open bars). (b) The results of MTT assay of Jurkat cells treated with **1** in the presence of Cu(NO_3_)_2_, Zn(NO_3_)_2_, FeSO_4_, Co(NO_3_)_2_, and NiSO_4_, respectively, ([**1**] = 0.78–25 μM and [M^2+^] = 1.71–37.5 μM) for 12 h (closed bars), 24 h (open bars), and 48 h (faded bars). (c) The results of MTT assay of Jurkat cells treated with **2** in the presence of Cu(NO_3_)_2_, Zn(NO_3_)_2_, FeSO_4_, Co(NO_3_)_2_, and NiSO_4_, respectively, ([**2**] = 0.78–25 μM and [M^2+^] = 1.71–37.5 μM) for 12 h (closed bars), 24 h (open bars), and 48 h (faded bars). (d) The results of MTT assay of Jurkat cells treated with BAPTA in the presence of Cu(NO_3_)_2_, Zn(NO_3_)_2_, FeSO_4_, Co(NO_3_)_2_, and NiSO_4_, respectively ([BAPTA] = 0.78–25 μM and [M^2+^] = 1.71–37.5 μM) for 24 h (open bars), and 48 h (closed bars). (e) The results of MTT assay of Jurkat cells treated with BAPTA-AM in the presence of Cu(NO_3_)_2_, Zn(NO_3_)_2_, FeSO_4_, Co(NO_3_)_2_, and NiSO_4_ for 1 h ([BAPTA-AM] = 0.78–25 μM and [M^2+^] = 1.71–37.5 μM) and then tested for 24 h (open bars) and 48 h (closed bars).

Next, we examined the effect of complexation of **1**, **2**, BAPTA, and BAPTA-AM with metal ions such as Cu(NO_3_)_2_, Zn(NO_3_)_2_, FeSO_4_, Co(NO_3_)_2_, and NiSO_4_ on their cytotoxicity against Jurkat cells. Mixture of ligands and metal ions were incubated for 1 h at 37 °C, and added to Jurkat cells for incubation for 12, 24, and 48 h at [ligand] = 0.78–25 μM and [M^2+^] = 1.71–37.5 μM ([ligand]: [M^2+^] = 1:1.5). After that, MTT assays were carried out, as shown in Figure S3 of the Supporting Information. Figure 9 summarizes the incubation time-dependent EC_50_ values of **1**, **2**, BAPTA, and BAPTA-AM against Jurkat cells (determined by MTT assays) in the presence of Cu(NO_3_)_2_, Zn(NO_3_)_2_, FeSO_4_, Co(NO_3_)_2_, and NiSO_4_ ([ligand]/[M^2+^] = 1:1.5), respectively, after the incubation for 12, 24, and 48 h (the results of the MTT assays of Jurkat cells in the presence of metal complexes of **1** and BAPTA with Zn^2+^, Ni^2+^, Co^2+^, Fe^2+^, and Cu^2+^ after the incubation for 24 h are displayed in Figure S3 in the Supporting Information).

Figure 9a shows cytotoxicity of metal ions, in which ligand-free Co^2+^ exhibits considerable toxicity (EC_50_ = 13 μM) after incubation for 48 h. Decrease in EC_50_ values of metal-free **1** and **2** (from ca. 9 to 0.06 μM for **1** and from ca. 2 to 0.1 μM for **2** after incubation for 12–48 h, respectively) suggests the time-dependent enhancement of anticancer activity of **1** and **2** themselves (Figure 9b,c, left). Weak–negligible effect of Zn^2+^ and Cu^2+^ on the cytotoxicity of **1** and **2** after incubation for 24 and 48 h in Figure 9b,c (please compare open bars and faded bars for **1** with no metal, Cu^2+^ and Zn^2+^) can be attributed to the kinetic instability of its Zn^2+^ and Cu^2+^ complexes, because the thermodynamic stability of the complexes of **1** with Zn^2+^, Ni^2+^, Co^2+^, Fe^2+^ and a Cu^2+^complexes is not so different, as described above. We do not exclude the possibility that tris(bpy) ligand **1** may function as Cu^2+^ carrier into the cancer cells, because the weak enhancement anticancer of **1** is observed in Figures 9b and S3 of the Supporting Information.

The EC_50_ value of **1**+Zn(NO_3_)_2_ (157 nM) after incubation for 24 h (open bars in Figure 9b) was nearly the same as that of **1** (137 nM). The EC_50_ values of **1** in the presence of Cu(NO_3_)_2_ (483 nM), Co(NO_3_)_2_ (>25 μM), NiSO_4_ (>25 μM), and FeSO_4_ (19.3 μM) were much greater (weaker cytotoxicity) than that of **1** alone. These data suggest that the reactivation of **1** and **2** (from OFF to ON) by decomplexation of their Zn^2+^, Co^2+^, and Fe^2+^ complexes is a time-dependent process. The EC_50_ values for the Ni^2+^-**1** and Co^2+^-**1** complexes were negligibly or weakly reduced even after 48 h, while the anti-cancer activity of Ni^2+^-**2** complex was somehow restored after 48 h (Figure 9c).

These data suggest the possibility that the biological activity of **1** and its analogs might be controlled by the complexation and decomplexation with biorelevant metal cations and/or by external ligands that affect the stability of these metal complexes. Consideration of the cytotoxicity of metal-free **1** and **2** (Table 1) and reactivation behaviors of their metal complexes after the incubation for 24–48 h suggests that combinations of **1** or **2** with Fe^2+^ would be the best for the suppression of the toxicity of these metal-free ligands and for the slow ON–OFF switching of anticancer activity (rightmost side of Figure 9b,c). The Zn^2+^-**1**, Cu^2+^-**1**, and Zn^2+^-**2** would be the next best candidates. It would be better to avoid the use of Ni^2+^ complexes of **1** and **2**, because the toxicity of Ni^2+^ has previously been reported.^48^

For comparison, BAPTA-AM showed a moderate anticancer activity against Jurkat cells and its EC_50_ values after the incubation for 24 and 48 h are almost identical (8.6–7.9 μM) (Figure S4 in the Supporting Information). The UV/vis absorption titrations of BAPTA-AM with Zn^2+^, Co^2+^, and Fe^2+^ show very small change (data not shown), indicating its very weak complexation between these metal ions. The results of UV/vis absorbance titrations of unmasked BAPTA with Zn^2+^, Ni^2+^, Co^2+^, Cu^2+^, and Fe^2+^ are shown in Figure S5 in the Supporting Information. The anticancer activity of BAPTA itself was weak, possibly due to its hydrophilicity (Figures 9d and S3b in the Supporting Information), and that of BAPTA-AM after incubation for 24 h and 48 h was enhanced by the complexation with Zn^2+^, Ni^2+^, Co^2+^, and Fe^2+^ (Figures 9e and S4 in the Supporting Information).

To investigate the mechanism responsible for the BAPTA-AM-induced cell death in Jurkat cells, Jurkat cells were incubated with Z-VAD-fmk (15 μM) for 3 h, and then cells were treated with BAPTA-AM (12.5 μM) for 24 h followed by an MTT assay. Figure S6 in the Supporting Information shows that Z-VAD-fmk considerably restored Jurkat cell viability after the treatment with BAPTA-AM, indicating the induction of apoptosis by BAPTA-AM.

### Crystal Structures of Metal-free 1 and Its Ni^2+^ Complex (**12**)

**Figure 10:**
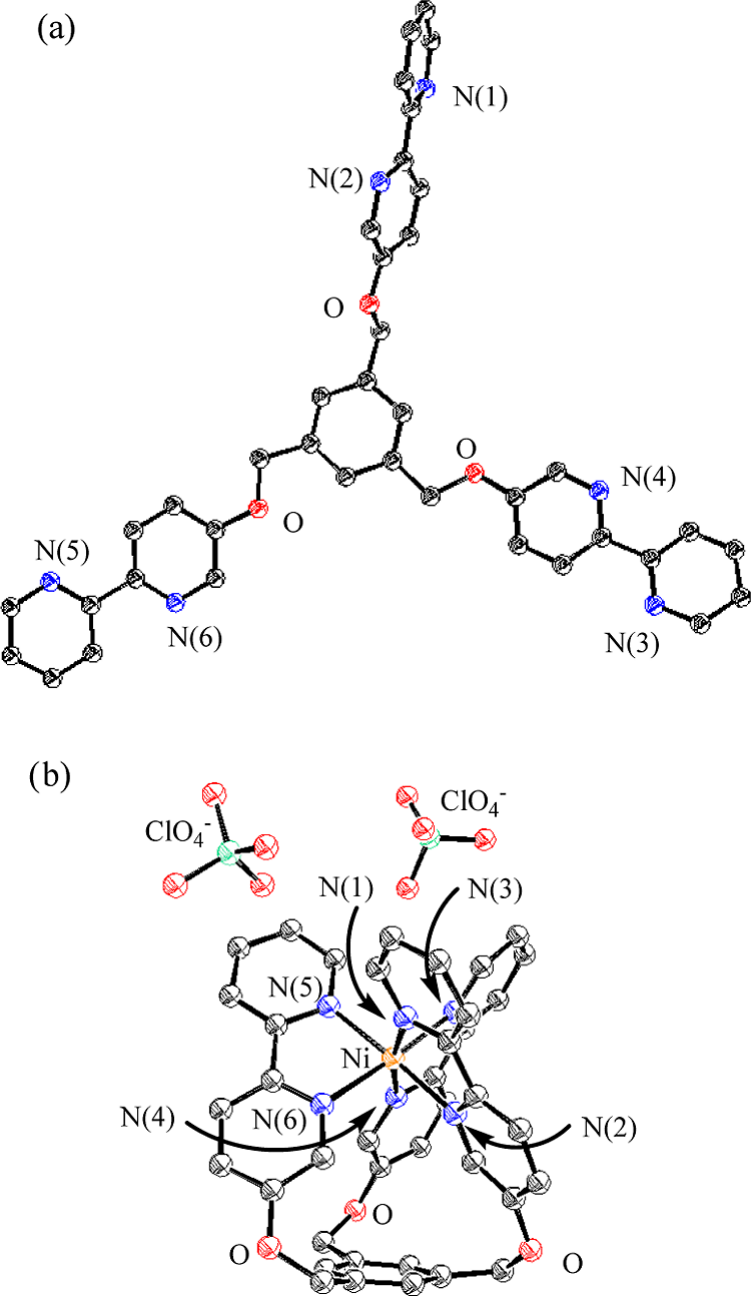
(a) ORTEP drawing of a crystal structure of metal-free **1** with ellipsoids set at 50% probability. (b) ORTEP drawing of a crystal structure of Ni complex of **1** (**12**) with ellipsoids set at 50% probability. Selected bond lengths [Å]: Ni–N(l) 2.065, Ni–N(2) 2.101, Ni–N(3) 2.084, Ni–N(4) 2.099, Ni–N(5) 2.080, Ni–N(6) 2.098. Acetonitrile in a crystal and hydrogen atoms have been omitted for clarity.

The X-ray single crystal structure of metal-free **1** is shown in Figure 10a, which confirms its “open form” structure, in which the three bpy units are extended horizontally with respect to the center benzene unit. In order to characterize the metal complexes of **1** and **2**, the crystallization of these ligands with Zn^2+^, Co^2+^, Cu^2+^, Ni^2+^, and Fe^2+^ was attempted and a good crystal was obtained for only the Ni^2+^ complex of **1**. As displayed in Figure 10b, the “closed form” of the Ni complex of **1** (**12**) was revealed by X-ray structure analysis, in which a Ni^2+^ cation adopts pseudo-octahedral 6-coordinated structure with coordination from six nitrogen atoms of **1** to the Ni^2+^ ion (the averaged value of six N–Ni coordination bonds is 2.09 Å) (typical parameters for X-ray crystal structure analysis of **1** and Ni^2+^-**1** (**12**) are summarized in Table S1 in the Supporting Information).

### Effect of Biorelevant Metal Ions on Cell Death of 1 against Jurkat Cells, as Examined by Ethidium Bromide/Acridine Orange Assays

**Figure 11:**
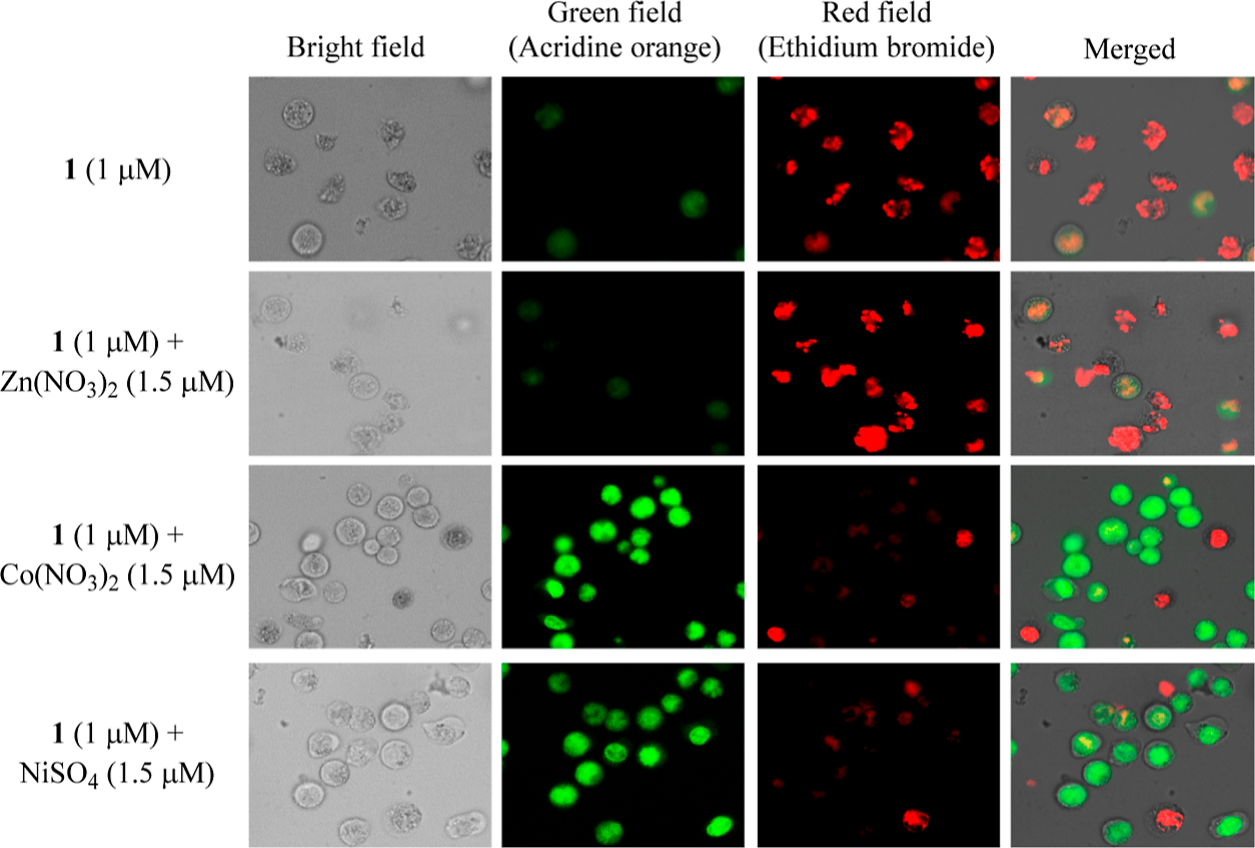
Jurkat cells were treated with **1** (1 μM) alone and complexes of **1** with Zn(NO_3_)_2_, Co(NO_3_)_2_ and NiSO_4_ for 24 h stained with acridine orange/ethidium bromide (AO/EB) and observed by using fluorescence microscopy. The samples were analyzed for green fluorescence (acridine orange) and red fluorescence (ethidium bromide).

The results of MTT experiments shown in Figure 9 indicate that Co(NO_3_)_2_ and NiSO_4_ inhibit Jurkat cell death induced by **1**. Furthermore, we conducted fluorescence microscopic analysis to check the effect of Co(NO_3_)_2_ (1.5 μM), NiSO_4_ (1.5 μM), and Zn(NO_3_)_2_ (1.5 μM) on cell death in Jurkat cells induced by **1** (1 μM). We observed a fluorescence emission from Jurkat cells after the treatment with **1** (1 μM) and then with ethidium bromide and acridine orange by fluorescence microscopy in which acridine orange, a nucleic acid binding dye, emits green fluorescence due to indication to nucleic acids, while ethidium bromide, is one of potent DNA intercalators, detects dead cells due to the DNA intercalation and the loss of membrane integrity.^49^ The different labeling patterns in this assay presented in Figure 11 allows live cells (green emission from acridine orange) and dead cells (red emission from ethidium bromide) to be identified, which suggests that the cell death induced by **1** is reduced considerably in the presence of Co^2+^ and Ni^2+^ cations.

### Change in the Concentrations of Fe^2+^ and Zn^2+^ during Apoptotic Processes Induced by **1**

As described in the Introduction, apoptosis induction by Fe^2+^ chelators such as tachpyr (Scheme 1) has been explained by their binding to intracellular Fe^2+^. Therefore, we examined the change in the concentrations of Fe^2+^ ions in Jurkat cells during apoptotic processes induced by **1** by using Mito-FerroGreen (a probe for labile Fe^2+^ in mitochondria) and FerroFarRed (a probe for Fe^2+^ in cells)^50,51^ (these structures are shown in Figure S7 in the Supporting Information). Jurkat cells (1.0 × 10^5^ cells/tube) were incubated at 37 °C for 24 h in the presence of **1** (0.5, 1 and 5 μM) and *N*,*N*,*N′*,*N′*-tetra(2-pyrydylmethyl)ethylene diamine (TPEN) (25 μM),^52^ a cell permeable, and potent metal (Zn^2+^ > Fe^2+^ > Mn^2+^) chelator, for comparison. Note that Mito-FerroGreen and FerroFarRed detect Fe^2+^ due to the reduction of their *N*-oxide moieties by Fe^2+^ and, hence, negligibly disturb the complexation of **1** with intracellular Fe^2+^.

**Figure 12:**
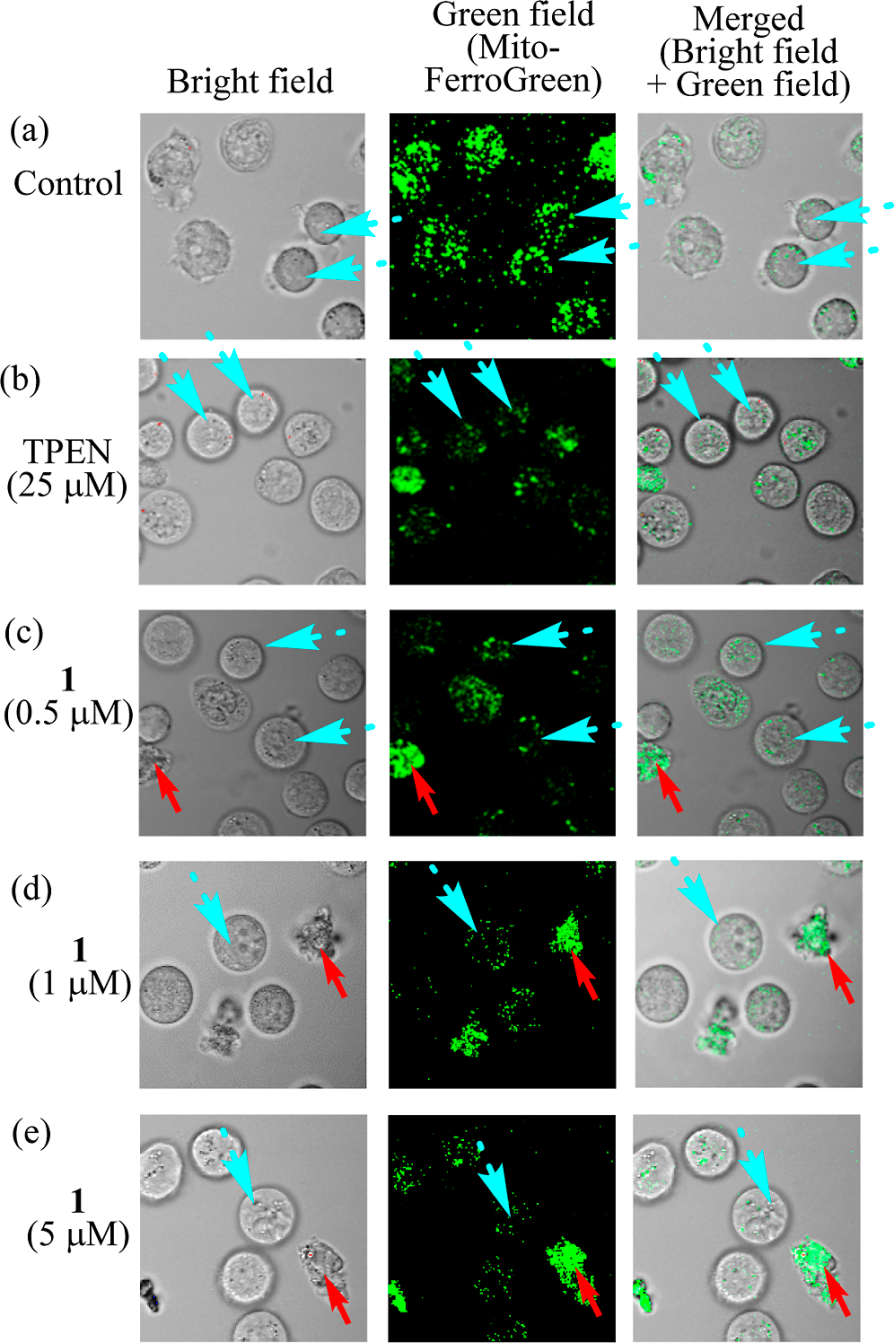
Staining of Jurkat cells with Mito-FerroGreen (5 μM) to detect Fe^2+^ ions in mitochondria. Jurkat cells were incubated with no ligand (control) (a), TPEN (25 μM) (b), **1** (0.5 μM) (c), **1** (1 μM) (d), and **1** (5 μM) (e) for 24 h, prior to the treatment with Mito-FerroGreen (left: bright field image, middle; green field image from Mito-FerroGreen, right: merged image). Red arrows indicate dead cells and light blue dashed arrows indicate live cell morphology.

**Figure 13:**
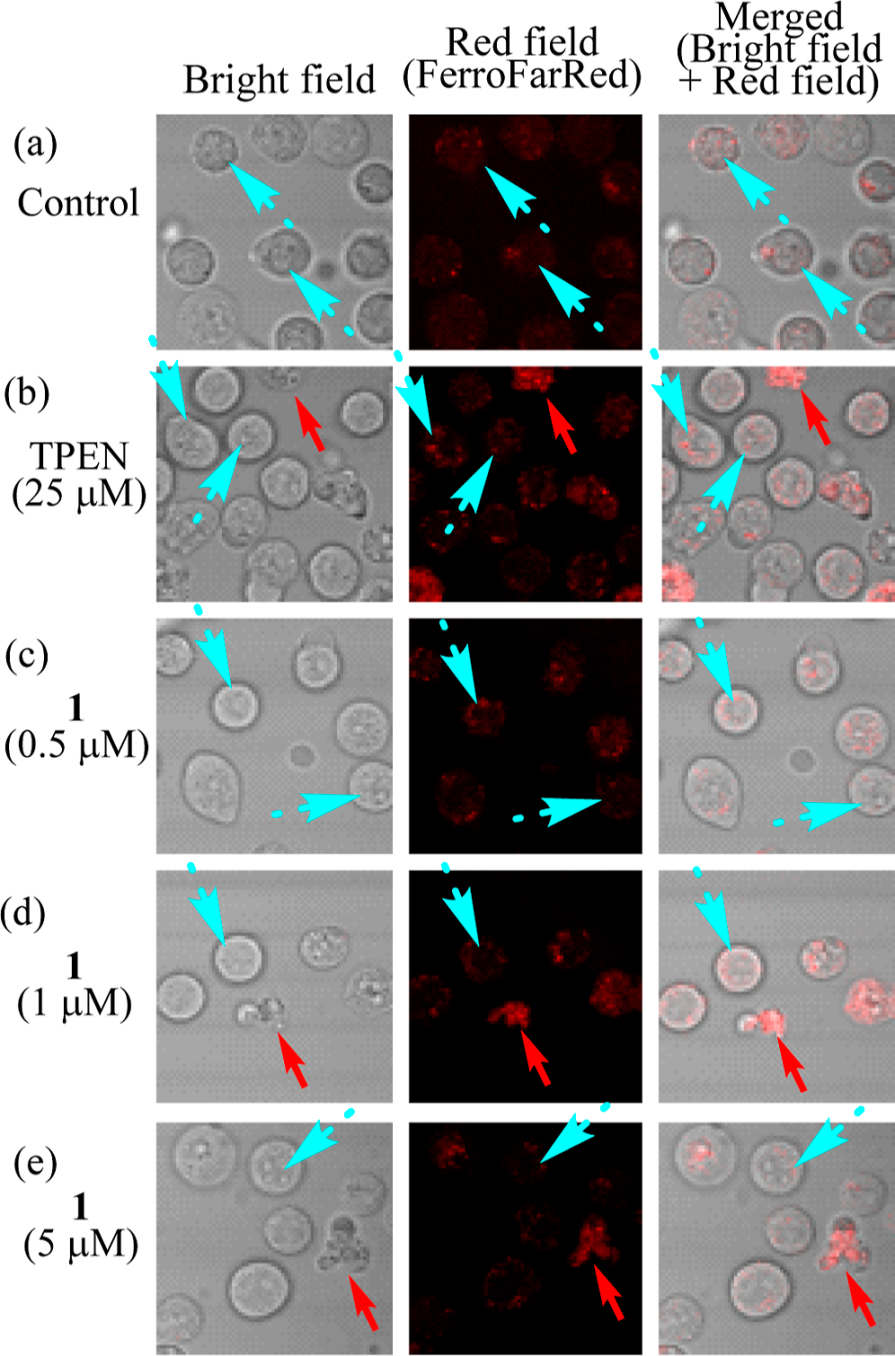
Staining of Jurkat cells with FerroFarRed (5 μM) to detect intracellular Fe^2+^ ions. Jurkat cells were incubated with no ligand (control) (a), TPEN (25 μM) (b), **1** (0.5 μM) (c), **1** (1 μM) (d), and **1** (5 μM) (e) for 24 h, prior to the incubation with FerroFarRed (left: bright field image, middle; red field image from FerroFarRed, right: merged image). Red arrows indicate dead cells and light blue dashed arrows indicate live cell morphology.

Green fluorescence images from Mito-FerroGreen (5 μM) in Figure 12 and red fluorescent images from FerroFarRed (5 μM) in Figure 13 suggest that the concentration of Fe^2+^ in mitochondria (indicated with light blue dashed arrows) is reduced rather than that in cytoplasm or in other intracellular organelle after the incubation with **1** (Figure 12c,d,e vs Figures 12a and Figure 13c,d,e vs Figure 13a). It should be mentioned that microscopic images of these Fe^2+^ in living cells were observed in Figures 12 and 13 even at [**1**] = 0.5–5 μM, which are close to its EC_50_ value (0.14 μM after incubation for 24 h, as listed in Table1), in order to observe the behaviors of Fe^2+^ in living cells (indicated with light blue dashed arrows) rather than in dead cells (indicated with red arrows). Figure 12b shows that cytotoxicity of TPEN is weaker than **1** even at 25 μM and that green emission from Mito-FerroGreen is negligibly–weakly reduced in the presence of TPEN, possibly because Fe^2+^ is trapped by TPEN.

In Figure 13, the red fluorescent signal was slightly increased in dead cells when metal chelators were present, suggesting that iron intake to cells is facilitated by the chelators. We do not exclude the possibility that **1** facilitates the production of reactive oxygen species (ROS) that oxidize Fe^2+^ to Fe^3+^, which cannot be detected by these Fe^2+^ fluorophores. The fact that more potent green and red emission is observed in dead cells, as indicated by red arrows in Figures 12d,e and 13d,e, may suggest the release of free Fe^2+^ in apoptotic cells.

It was described that the intracellular concentration of free Zn^2+^ is enhanced in the early stage of apoptosis by using Zn^2+^ fluorophores such as zinquin (its structure is shown Figure S7 in the Supporting Information)^53,54^ and so on.^55,56^ Therefore, the change in the concentrations of Zn^2+^ was checked by zinquin (25 μM). Figure S8 in the Supporting Information suggests that the concentration of Zn^2+^ was decreased in living cells after the treatment with **1** (indicated with light blue arrows) and then enhanced in dead cells (indicated with red arrows). Because it is difficult to discriminate the early stage and late stage of **1**-induced apoptosis, we would like to mention the possibility that intracellular free Zn^2+^ ions are trapped by **1** to stimulate apoptotic pathways and that Zn^2+^ ions are released at early stage or middle–late stages of apoptosis, although its details are yet to be studied.

### Possible Scheme of the Regulation of Cytotoxicity of **1** in Jurkat Cells

**Scheme 6:**
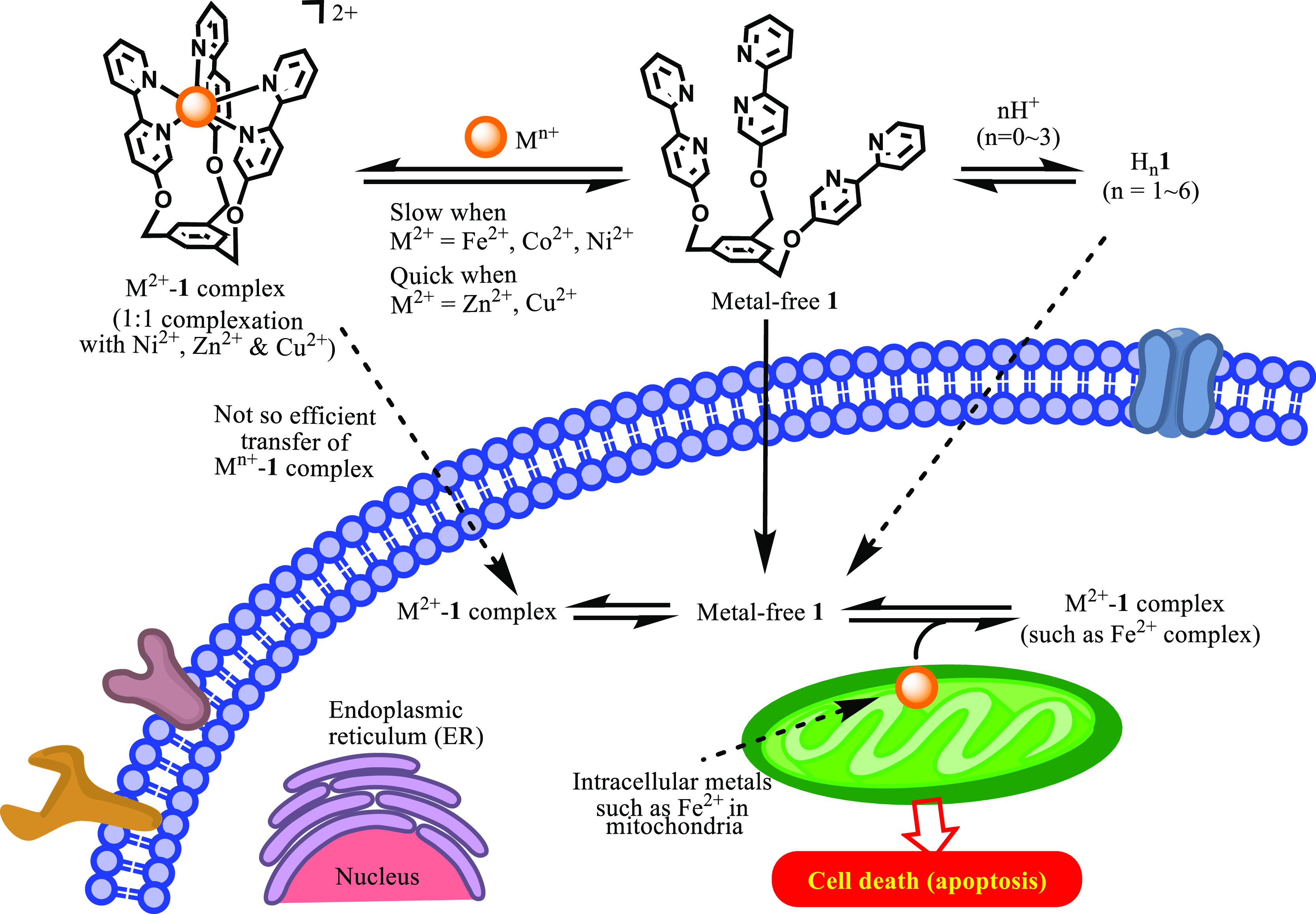
Proposed Mechanism of Apoptosis-Inducing Activity of **1** and Its Metal Complexes

Based on the aforementioned results, a proposed scheme for the apoptosis induced by **1** is presented in Scheme 6. The tris(bpy) ligand **1** induces apoptosis against various types of cancer cells including Jurkat and A549 cells and has a weak cytotoxicity against IMR-90. This cytotoxicity is strongly inhibited by the addition of metals, indicating the metal complexes of **1** have a low cell-membrane permeability. The stable complexes of **1** with Ni^2+^ and Co^2+^ have only weak cytotoxicity and its Zn^2+^, Cu^2+^, and Fe^2+^ complexes are reactivated in a time-dependent manner, suggesting that the decomplexation results in the release of metal-free **1**, which is transferred into cancer cells, traps intracellular metals, and induces apoptosis. Namely, the anticancer activity of poly(bpy) ligand complexes is turned OFF by their complexation with metals and is turned ON by the decomplexation. It is likely that most possible target of **1** is Fe^2+^ in mitochondria, although the effect on the concentrations of intracellular Zn^2+^, Cu^2+^, and other metal ions cannot be excluded. These results may suggest that poly(bpy) ligands may be potent drug candidates for the treatment of cancer and related diseases and that their side effect might be controlled (reduced) by the complexation with appropriate metals. It is suggested that Fe^2+^ complex **1** and **2** would be the best candidates in this work and the next best would be Zn^2+^-**1**, Cu^2+^-**1**, and Zn^2+^-**2** complexes, as described above.

## Conclusions

In this study, we report on the design and synthesis of poly(bpy) ligands as novel chelating agents for use in inducing cell death of cancer cells. We examined the metal complexation properties and conducted biological evaluation of these poly(bpy) ligands. The results of MTT assays revealed that tris(bpy) **1** and bis(bpy) **2** exhibit a more potent cytotoxicity against blood cells (Jurkat, MOLT-4, U937 cells) than mono(bpy) **3** and are more potent against these cancer cell lines than against IMR-90, a model of normal cell. The results of Annexin V/PI staining and Western blot experiments suggest that these derivatives induce apoptotic cell death with the cleavage of caspase-3 and a flip-flop of the cell membrane. A suppressive effect of various metal ions on the cytotoxicity of **1** against Jurkat cells was also observed in MTT assays. It has been described that metal chelators used for chelation therapy have multiple molecular targets and act by various mechanisms with several side effects related to its use, including myelo-suppression, hypoxia, and methemoglobinemia. The results described in this manuscript suggest the possibility that the biological activity and side effects of metal chelators such as **1** and its analogs could be controlled by complexation and decomplexation with intracellular metal cations. To date, considerable examples of metal complexes of bpy-type ligands have been reported.^57^ The results described in this manuscript may afford important information for the future design and synthesis of bpy-type anticancer agents.

We previously reported that hybrid compounds of cyclometalated iridium(III) (Ir(III)) complexes with cyclic peptides that bind to death receptors (DRs) expressed on the cancer cells, which detect Jurkat cells and induce their apoptosis and necrosis-type cell death.^58,59^ In addition, Ir(III) complexes and triptycene compounds conjugated with cationic peptides such as KK(K)GG (K: lysine, G: glycine) sequences, which induce paraptosis, a non-apoptotic programmed cell death, in Jurkat cells and other cancer cell lines, were designed and synthesized.^58−61^ Therefore, our future efforts will be directed to the design and synthesis of hybrid compounds of poly(bpy) ligands with DR-binding peptides and/or cationic peptide units, which would give the answers to the following question, which will be the dominant type of cell death, apoptosis and/or paraptosis, or other types.

## Experimental Procedures

### General Information

All reagents and solvents were purchased at the highest commercial quality and were used without further purification. Anhydrous DMF was obtained by distillation from calcium hydride. All aqueous solutions were prepared using deionized and distilled water. Melting points were measured using a Yanaco MP-J3 Micro Melting Point apparatus and are uncorrected. UV/vis absorption spectra were recorded on a JASCO V-630 spectrophotometer at 37 °C. The UV/vis absorption titrations of **1** with metal ions were carried out by addition of 10 mM solutions of Zn(NO_3_)_2_, NiSO_4_, Co(NO_3_)_2_, Fe(NO_3_)_2_, and Al(NO_3_)_3_ in H_2_O and 10 mM solutions of Cu(NO_3_)_2_ in DMSO. The apparent stability constants of metal–ligand complexes (*K*_s_) were calculated by using the Bind Works 1.0 software provided by CSC (Calorimetry Science Corp.), an interactive PC software for the analysis of Binding Isotherms on Isothermal Titration Calorimeters (ITCs), to which the change in absorption values obtained by the UV/vis absorption titrations are input.^62^ IR spectra were measured with a PerkinElmer FT-IR spectrophotometer (Spectrum 100) at room temperature. ^1^H (300 and 400 MHz) NMR spectra were recorded on a JEOL Always 300 spectrometer and a JEOL Always 400 spectrometer, respectively. Luminescence imaging studies were performed using a fluorescent microscope (Biorevo, BZ-9000, Keyence). Tetramethylsilane (TMS) was used as an internal reference for ^1^H NMR measurements in CDCl_3_ and DMSO-*d*_6_. Mass spectral measurements were performed on a JEOL JMS-SX102A and Varian TQ-FT. Thin-layer chromatography (TLC) and silica gel column chromatography were performed using Merck Art. 5554 (silica gel) TLC plates and Fuji Silysia Chemical FL-100D, respectively. MTT was purchased from Dojindo. Z-VAD-fmk was purchased from Peptide Institute and ethidium bromide was purchased from Nacalai tesque. Monothioglycerol (MTG) was purchased from WAKO Pure Chemical Industries. Propidium iodide (PI) and AnnexinV were purchased from Invitrogen. Caspase-3 was purchased from Santa Cruz Biotechnology, USA, and GAPDH was purchased from Cell Signaling Technology, USA.

#### Synthesis: 5′-(Benzyloxy)-2,2′-bipyridine 1-Oxide (**8**)

Pyridine *N*-oxide **7**(30) (8.2 g, 86.7 mmol), 5-(benzyloxy)-2-bromopyridine **5**(29) (7.6 g, 28.7 mmol), Pd(OAc)_2_ (323.3 mg, 1.44 mmol), and K_2_CO_3_ (8.0 g, 57.8 mmol), abd P(^*t*^Bu)_3_ (405 μL, 1.72 mmol) were reacted in anhydrous toluene (150 mL) under argon atmosphere at 110 °C for 4 days. The product was filtrated off by using filter paper and purified by silica gel column chromatography (hexanes/acetone eluent with a gradient composition of 20–33 vol % for acetone), which yielded 3.20 g (11.5 mmol, 40%) of **8** as a yellow solid. mp 50–52 °C. IR (ATR): ν = 1588, 1450, 1382, 1229, 1114, 1008, 842, 735, 712, 696, 626, 416 cm^–1^. ^1^H NMR (300 MHz, CDCl_3_/TMS): δ = 9.00 (d, *J* = 6.6 Hz, 1H), 8.48 (d, *J* = 2.1 Hz, 1H), 8.29 (dd, *J* = 4.8, 0.6 Hz, 1H), 8.20 (ddd, *J* = 5.6, 1.5, 1.0 Hz, 1H), 7.44–7.36 (m, 7H), 7.44–7.36 (m, 1H), 5.19 (s, 2H), ^13^C NMR (100 MHz, CDCl_3_/TMS): δ = 155.2, 147.1, 142.0 140.7, 138.5, 135.8, 128.8, 128.4, 127.6, 127.3, 126.2, 125.7, 124.5, 120.5, 70.4 ppm. ESI-MS (*m*/*z*): calcd for C_17_H_14_N_2_O_2_ [M + H]^+^, 279.11297; found, 279.11280.

#### 5-Hydroxy-2,2′-bipyridine (9)

A solution of **8** (278.8 mg, 1.0 mmol) and 5% Pd–C (80.0 mg) in MeOH (6 mL) was degassed (×3) and stirred at room temperature for 3 days under H_2_-atmosphere (1 atm). The solution was filtered through celite, and the solvent was removed in vacuum to yield 276.6 mg (1.6 mmol, quant.) of **9** as a yellow solid without further work-up. mp 89–91 °C. IR (ATR): ν = 2961, 2920, 1573, 1439, 1269, 845, 790, 746, 584 cm^–1^. ^1^H NMR (400 MHz, CD_3_OD/TMS): δ = 8.66 (d, *J* = 3.6 Hz, 1H), 8.38 (d, *J* = 3.0 Hz, 1H), 8.31 (d, *J* = 6.6 Hz, 1H), 8.29 (d, *J* = 4.5 Hz, 1H), 7.82 (td, *J* = 6.0, 1.2 Hz, 1H), 7.36 (d, *J* = 2.1 Hz, 1H), 7.33 (d, *J* = 1.8 Hz, 1H) ppm. ^13^C NMR (100 MHz, CDCl_3_/TMS): δ = 158.9, 158.9, 152.3, 150.0, 142.1, 141.0, 128.3, 127.3, 126.7, 124.9 ppm. ESI-MS (*m*/*z*): calcd for C_10_H_8_N_2_O [M + H]^+^, 173.0701; found, 173.0794.

#### 1,3,5-Tris(([2,2′-Bipyridin]-5-yloxy)methyl)benzene (1)

A mixture of **9** (243.7 mg, 0.83 mmol), 1,3,5-tris(bromomethyl)benzene **10** (95.1 mg, 0.27 mmol), K_2_CO_3_ (516.4 mg, 3.7 mmol), and tetra-*n*-butylammonium iodide (TBAI) (3.3 mg, 0.085 mmol) in dist. DMF (1.0 mL) was stirred at room temperature for 1 day. The product was filtrated on the filter paper and purified by NH silica gel column chromatography (hexanes/ethyl acetate eluent with gradient composition of 9–11 vol % for ethyl acetate) yielded 69.6 mg (0.11 mmol, 42%) of **1** as a colorless solid. mp 94–95 °C. IR (ATR): ν = 1573, 1449, 1436, 1255, 1217, 835, 790, 745, 736, 633, 405 cm^–1^. ^1^H NMR (300 MHz, CDCl_3_/TMS): δ = 8.63 (d, *J* = 3.6 Hz, 3H), 8.45 (d, *J* = 2.1 Hz, 3H), 8.35 (d, *J* = 6.6 Hz, 3H), 8.30 (d, *J* = 6.0 Hz, 3H), 7.80–7.78 (m, 3H), 7.55 (s, 3H), 7.41–7.38 (m, 4H), 5.24 (s, 6H) ppm. ^13^C NMR (100 MHz, CDCl3/TMS): δ = 155.9, 155.0, 149.4, 149.1, 137.5, 137.4, 136.9, 126.3, 123.0, 122.1, 121.8, 120.5, 70.0 ppm. ESI-MS (*m*/*z*): calcd for C_39_H_30_N_6_O_9_ [M + H]^+^, 653.22657; found, 653.22716. Anal. Calcd for C_39_H_30_N_6_O_3_ + H_2_O + 0.5EtOAc: C, 71.08; H, 5.24; N, 12.13%. Found: C, 70.79; H, 4.99; N, 11.76%.

#### 1,3-Bis(([2,2′-Bipyridin]-5-yloxy)methyl)benzene (2)

A mixture of **9** (113.5 mg, 0.66 mmol), 1,3-bis(bromomethyl)benzene **11** (79.3 mg, 0.30 mmol), K_2_CO_3_ (589.2 mg, 4.3 mmol), and TBAI (6.6 mg, 0.018 mmol) in dist. DMF (1.0 mL) was stirred at room temperature for 2 days. The product was filtrated off on a paper filter and purified by silica gel column chromatography (1% ammonia hexanes/ethyl acetate eluent with gradient composition of 33–50 vol %), which yielded 45.6 mg (0.18 mmol, 81%) of **2** as a colorless solid. mp 135–136 °C. IR (ATR): ν = 1572, 1452, 1256, 1219, 1015, 994, 846, 790, 745, 629, 403 cm^–1^. ^1^H NMR (300 MHz, CDCl_3_/TMS): δ = 8.64 (dd, *J* = 2.9, 0.9, 2H), 8.45–8.44 (m, 2H), 8.34 (dd, *J* = 6.6, 0.3 Hz, 2H), 8.30 (dd, *J* = 6.0, 0.6 Hz, 2H), 7.78 (td, *J* = 6.0, 1.5, 2H), 7.57 (s, 1H), 7.46 (s, 2H), 7.39 (ddd, *J* = 6.5, 2.4, 2.1 Hz, 3H), 5.21 (s, 4H) ppm. ^13^C NMR (100 MHz, CDCl_3_/TMS): δ = 156.0, 155.2, 149.4, 149.1, 137.6, 136.9, 136.8, 129.3, 127.5, 126.6, 123.1, 122.2, 121.8, 120.5, 70.3 ppm. ESI-MS (*m*/*z*): calcd for C_28_H_22_N_4_O_6_ [M + H]^+^, 447.18074; found, 447.18155. Anal. Calcd for C_28_H_22_N_4_O_2_ + 0.6H_2_O: C, 73.54; H, 5.11; N, 12.25%. Found: C, 73.52; H, 4.89; N, 11.92%.

#### 5-(Benzyloxy)-2,2′-bipyridine (3)

A mixture of **9** (37.8 mg, 0.22 mmol), benzyl bromide (57.2 mg, 0.33 mmol), and K_2_CO_3_ (94.3 mg, 0.68 mmol) in dist. DMF (1.5 mL) was stirred at 70 °C for 14.5 h. The product was filtrated and purified by silica gel column chromatography (1% ammonia hexane/ethyl acetate eluent with gradient composition of 25–33 vol %), which yielded 45.6 mg (0.18 mmol, 81%) of **8** as a colorless solid. mp 85–87 °C. IR (ATR): ν = 1572, 1558, 1451, 1254, 994, 845, 762, 729, 693, 630, 406 cm^–1^. ^1^H NMR (300 MHz, CDCl_3_/TMS): δ = 8.64 (d, 3.6 Hz, 1H), 8.44 (d, *J* = 3.0 Hz, 1H), 8.34 (d, *J* = 8.7 Hz, 1H), 8.30 (d, *J* = 7.8 Hz, 1H), 7.78 (ddd, *J* = 7.8, 7.5, 1.8 Hz, 2H), 7.60–7.37 (m, 6H), 5.18 (s, 2H) ppm. ^13^C NMR (100 MHz, CDCl_3_/TMS): δ = 156.1, 155.3, 149.3, 149.1, 137.7, 136.9, 136.1, 128.8, 128.4, 127.7, 123.0, 122.1, 121.8, 120.5, 70.5 ppm. ESI-MS (*m*/*z*): calcd for C_17_H_14_N_2_O [M + H]^+^, 263.11716; found, 263.11789. Anal. Calcd for C_17_H_14_N_2_O + 0.3H_2_O: C, 76.27; H, 5.50; N, 10.46%. Found: C, 76.04; H, 5.11; N, 10.12%.

### Single Crystal X-ray Diffraction Study of Metal-free 1 and Ni Complex of **1** (12)

The single crystal X-ray diffraction study of the metal-free **1** and Ni complex of **1** (**12**) was carried out using a Rigaku X-ray diffractometer equipped with a molybdenium MicroMax-007 and Saturn 724+ detector. The structure was solved by the direct method (SHLEXT) and refined by full-matrix least-squares methods on F2 using Olex2-1.3 program. Geometrical restraints, i.e., AFIX and OMIT were used in the refinements. For the metal-free **1**, SQUEEZE was used for the refinement because of the solvent disorder. All non-hydrogen atoms were refined anisotropically in the structure. The crystal data reported herein can be obtained free of charge from The Cambridge Crystallographic Data Centre via https://www.ccdc.cam.ac.uk/data_request/ cif. Crystal data and structure refinement for metal-free **1** (CCDC deposit number: 2062877) and **12** (Ni complex of **1**) (CCDC deposit number: 2062878) were described in the Supporting Information (Table S1 in the Supporting Information).

Crystals of metal-free **1** and its Ni complex **12** suitable for X-ray analysis were obtained by the recrystalization from CH_3_CN solution at room temperature. The measurement was performed at 123 K. The representative crystal data are as follows: For metal-free **1**, formula = C_78_H_60_N_12_O_6_, FW = 1261.38, triclinic, space group *P*1̅, *a* = 9.925(5), *b* = 17.269(8), *c* = 20.8607(10) Å, α = 82.865(16), β = 88.492(17), γ = 88.011(16), *V* = 3545(2) Å^3^, *Z* = 2, *T* = 123 K *D*_calcd_ = 1.182 g cm^–3^, μ(Mo Kα) = 0.077 cm^–1^, 2θ_max_ = 27.5^°^, λ(Mo Kα) = 0.71075 Å, 28,933 reflections measured, 15,530 unique, 5215 (*I* > 2σ(*I*)) were used to refine 865 parameters, 0 restraints, *wR*_2_ = 0.2937, *R*_1_ = 0.1148 (*I* > 2σ(*I*)), GOF = 0.954. A total of 28,933 reflections were collected, among which 5423 reflections were independent (*R*_int_ = 0.1247). The reasons and comments for B-alerts of **1** are described in Table S1 of the Supporting Information.

For Ni-**1** complex (**12**), formula = C_82_H_66_Cl_4_N_14_Ni_2_O_22_, FW = 1858.70, monoclinic, space group *P*21/*n*, *a* = 23.755(3), *b* = 14.3713(18), *c* = 24.465(3) Å, α = 90, β = 112.091(2), γ = 90, *V* = 7739.0(17) Å^3^, *Z* = 4, *T* = 123 K *D*_calcd_ = 1.595 g cm^–3^, μ(Mo Kα) = 0.715 cm^–1^, 2θ_max_ = 27.5^°^, λ(Mo Kα) = 0.71075 Å, 62,388 reflections measured, 17,696 unique, 13,123 (*I* > 2σ(*I*)) were used to refine 1119 parameters, 0 restraints, *wR*_2_ = 0.1521, *R*_1_ = 0.0646 (*I* > 2σ(*I*)), GOF = 1.059. A total of 62,388 reflections were collected, among which 5217 reflections were independent (*R*_int_ = 0.1247). The reasons and comments for B-alerts of **12** are described in Table S1 of the Supporting Information.

### Cell Culture

Jurkat, MOLT-4, and U937cell lines were cultured in RPMI 1640 medium supplemented with 10% heat-inactivated fetal bovine serum (FBS), l-glutamine, HEPES (2-[4-(2-hydroxyethyl)-1-piperazinyl]ethanesulfonic acid, p*K*_a_ = 7.5), penicillin/streptomycin, and monothioglycerol (MTG) in a humidified 5% CO_2_ incubator at 37 °C. A549 cells and IMR-90 cells were grown in DMEM (low glucose) supplemented with 10% FBS, l-glutamine, and penicillin/streptomycin under 5% CO_2_ at 37 °C. HeLa S3 cells were grown in MEM supplemented with 10% FBS, l-glutamine, and penicillin/streptomycin under 5% CO_2_ at 37 °C.

### MTT Assay

(a) Cytotoxic study. Jurkat cells, MOLT-4 cells, and U937 cells (1.5 × 10^5^ cells/mL per one well) were incubated in 10% FBS RPMI 1640 medium containing **1** (0–25 μM), **2** (0–25 μM) , **3**, cisplatin (0–100 μM), and actinomycin D (0–10 μM) under 5% CO_2_ at 37 °C for 24 h on 96 well plates (Watson), 0.5% MTT (3-(4,5-dimethyl-2-thiazolyl)-2,5-diphenyl-2*H*-tetrazolium bromide) reagent in PBS (10 μL) was then added to the cells. After incubation at 37 °C for 4 h, a formazan lysis solution (10% SDS in 0.01 N HCl) (100 μL) was added and the resulting solution was incubated overnight under the same conditions, followed by the measurement of absorbance at 570 nm with a microplate reader (BIO-RAD).

HeLa S3 cells and A549 cells in MEM and DMEM medium with 10% FBS (1.5 × 10^5^ cells/mL per one well) were seeded in to 96 well plates and incubated for 24 h, MTT assay of poly(bpy) ligands (0–25 μM), cisplatin (0–100 μM), and actinomycin D (0–10 μM) was performed according to the same procedure described above.

IMR-90 cells in DMEM medium with 10% FBS (1.5 × 10^5^ cells/mL) were seeded in to 96 well plates and incubated for 24 h at 37 °C, MTT assays with poly(bpy) ligands and their metal complexes (0–100 μM), cisplatin (0–100 μM), and actinomycin D (0–10 μM) were performed following the same procedure described above. These experiments were carried out at least three times.

(b) MTT assay in the presence of a caspase inhibitor. Jurkat cells were pre-treated with Z-VAD-fmk (15 μM) for 3 h, a general caspases inhibitor, followed by the treatment with **1** (1 μM), BABTA-AM (12.5 μM) and cisplatin (50 μM), respectively, for 24 h, and then MTT assay was carried out as described above.
(c) For evaluation of the effect of metal ions on the cytotoxicity of **1**, **2**, BAPTA, and BAPTA-AM, these ligands were incubated with metal ions such as Cu(NO_3_)_2_, Zn(NO_3_)_2_, FeSO_4_, Co(NO_3_)_2_, and NiSO_4_ for 1 h, respectively, and added to Jurkat cells. The whole mixtures were incubated for 12, 24, and 48 h at [ligand] = 0.78–25 μM and at [M^2+^] = 1.71–37.5 μM ([ligand]/[M^2+^] = 1:1.5). The MTT assays were conducted as described above.

### Western Blot Analysis

Jurkat cells (3.0 × 10^6^ cells) were incubated with **1** (0–20 μM) and cisplatin (0–50 μM) for 24 h under 5% CO_2_ at 37 °C. After the treatment, cells were washed twice with ice cold PBS and proteins were extracted in RIPA buffer (Nacalai Tesque, Japan). The extracted proteins were quantified by using a Pierce BCA Protein Assay Kit (Thermo Scientific). Proteins at 50 μg/well were used for SDS-PAGE (7.5–15%) (BioRad, USA). After SDS-PAGE, the gel was transferred to polyvinylidene fluoride membrane (Merck Millipore, Germany) using semi dry blotter (BioRad, USA). The membrane was blocked with Blocking One solution (Nacalai Tesque, Japan) for 30 min at room temperature. After blocking, membrane was washed three times with TBST (5 min at each time) and incubated overnight with primary antibodies diluted in signal enhancer HIKARI- solution A (Nacalai Tesque, Japan). The next day, the membrane was washed three times with TBST and incubated for 1 h at r.t. with a secondary antibody such as anti-rabbit or anti-mouse diluted in signal enhancer HIKARI-solution B (Nacalai Tesque, Japan). The protein signal was spotted by Chemi-Lumi One Ultra solution (Nacalai Tesque, Japan) using ChemiDoc MP system (BioRad, USA).

### Annexin V-FITC/PI Staining Assays by Flow Cytometry

Jurkat cells (1.0 × 10^5^ cells/tube) were incubated in 10% FBS RPMI 1640 medium containing solution of **1** (10^5^ μM) and cisplatin (0–50 μM) for 24 h. The cells were then centrifuged at 3000 rpm for 5 min at 4 °C, then the supernatant was discarded, and the pellet was resuspended in 200 μL of PBS. The cells were then centrifuged at 3000 rpm for 5 min at 4 °C, then the supernatant was discarded, and the pellet was resuspended in 195 μL of 1 × binding buffer. A 195 μL aliquot of the sample solution was incubated with 5 μL of FITC-conjugated annexin V (Invitrogen) for 15 min at room temperature in the dark. Then the cells were then washed by 1 × binding buffer and added 190 μL of 1 × binding buffer 10 μL of PI (20 μg/mL, Invitrogen). 200 μL of the sample solution transferred to culture tube were analyzed by flow cytometry (Becton Dickinson) using Cell Quest Research Software (Becton Dickinson).

### Annexin V-FITC/PI Stained Assays by Fluorescence Microscopy

Jurkat cells (1.0 × 10^5^ cells/tube) were incubated in 10% FBS RPMI 1640 medium containing solution of **1** (1 μM) and cisplatin (50 μM) for 24 h. The cells were centrifuged at 3000 rpm for 5 min at 4 °C, then the supernatant was discarded, and the pellet was resuspended in 200 μL of PBS. The cells were then centrifuged at 3000 rpm for 5 min at 4 °C, then the supernatant was discarded, and the pellet was resuspended in 195 μL of 1 × binding buffer. A 195 μL of the sample solution was incubated with 5 μL of FITC-conjugated annexin V (Invitrogen) for 15 min at room temperature in the dark. Then the cells were then washed with binding buffer (1 ×) and treated with 190 μL of binding buffer (1×) and 10 μL of PI (20 μg/mL, Invitrogen). A 200 μL of aliquot of the sample solution was transferred to a culture tube, and observed by fluorescent microscopy (excitation 540 nm, emission 605 nm, TRITC filter and excitation 502 nm, emission 526 nm, GFP-B filter).

### Acridine Orange/Ethidium Bromide Staining by Fluorescence Microscopy

Jurkat cells (1.0 × 10^5^ cells/tube) were incubated in 10% FBS RPMI 1640 medium containing solution of **1** (1 μM) with Zn(NO_3_)_2_ (1.5 μM), Co(NO_3_)_2_ (1.5 μM), and NiSO_4_ (1.5 μM) ions for 24 h. Then the cells were washed with PBS twice, and 100 μL of PBS and 10 μL of acridine orange/ethidium bromide was added. Then sample solution was incubated for 15–20 min at room temperature in the dark and observed by fluorescent microscopy (excitation 540 nm, emission 605 nm, TRITC filter and excitation 502 nm, emission 526 nm, GFP-B filter).

### Detection of Fe^2+^ and Zn^2+^ Ions in Cancer Cells by Fluorescence Microscopy

Jurkat cells (1.0 × 10^5^ cells/tube) were incubated at 37 °C for 24 h in the presence of **1** (0.5, 1, and 5 μM) and TPEN (25 μM), a cell permeable and potent metal (Zn^2+^ > Fe^2+^ > Mn^2+^) chelator, and stained with Mito-FerroGreen (a probe for labile Fe^2+^ in mitochondria) (5 μM), FerroFarRed (a probe for Fe^2+^ in cells) (5 μM)and zinquin (a probe for intracellular Zn^2+^) (25 μM). Then cell images were captured by using fluorescent microscopy.

## Supporting Information Available

Crystal data and structure refinement for **1** and **12**; the results of MTT assays of A549, HeLa S3, U937, MOLT-4, and IMR-90 cells after the incubation with BAPTA and BAPTA-AM; the results of UV/vis absorption titrations of **1** with Zn^2+^, Ni^2+^, Co^2+^, Cu^2+^, Fe^2+^, and Al^3+^; the results of MTT assays of Jurkat cells treated with **1** and BAPTA for 24 h in the presence of Ni^2+^, Co^2+^, Fe^2+^, Zn^2+^, and Cu^2+^; the results of MTT assays of Jurkat cells treated with BAPTA-AM in the presence of Ni^2+^, Co^2+^, Fe^2+^, Zn^2+^, and Cu^2+^; the results of UV/vis absorption titrations of BAPTA with Zn^2+^, Ni^2+^, Co^2+^, and Cu^2+^; effect of Z-VAD-fmk on the cell death in Jurkat cells induced by BAPTA-AM; the results of MTT assays of decomplexation of the Ni^2+^-**1** and Co^2+^-**1** complexes by using EDTA on the cytotoxicity of **1**; structures of Mito-FerroGreen, FerroFarRed, and zinquin; and staining of Jurkat cells with zinquin to detect intracellular Zn^2+^ ions (PDF)
