# Supplementary material for: Design and Synthesis of Poly(2,2′-Bipyridyl) Ligands for Induction of Cell Death in Cancer Cells: Control of Anticancer Activity by Complexation/Decomplexation with Biorelevant Metal Cations
Source: Inorg Chem. 2023 Aug 29;62(36):14615–31. doi: 10.1021/acs.inorgchem.3c01738 (PMC10498496; doi:10.1021/acs.inorgchem.3c01738)
Supplement: Supplementary file 1 — ic3c01738_si_001.pdf [file ic3c01738_si_001.pdf]

Supporting Information

Design and Synthesis of Poly(2,2'-bipyridyl) Ligands for Induction of Cell Death in Cancer Cells: Control of Anticancer Activity by Complexation/Decomplexation with Biorelevant Metal Cations

Chandrasekar Balachandran,[a,b] Masumi Hirose,[a] Tomohiro Tanaka,[a] Jun Jie Zhu,[a]

Kenta Yokoi,[a] Yosuke Hisamatsu,[a,c] Yasuyuki Yamada,[d,e] and Shin Aoki,[,a,b,f]*

[a]Faculty of Pharmaceutical Sciences, Tokyo University of Science, 2641 Yamazaki, Noda, 278-8510, Japan

[b]Research Institute for Biomedical Sciences, Tokyo University of Science, 2641 Yamazaki, Noda, Chiba 278-8510, Japan

[c]Graduate School of Pharmaceutical Sciences, Nagoya City University, 3-1 Tanabe-dori, Nagoya, Aichi 467-8603, Japan

[d]Department of Chemistry, Graduate School of Science, Nagoya University, Furo-cho, Chikusa-ku, Nagoya 464-8602, Japan

[e]Research Center for Materials Science, Nagoya University, Furo-cho, Chikusa-ku, Nagoya 464-8602, Japan

[f]Research Institute for Science and Technology, Tokyo University of Science, 2641 Yamazaki, Noda, Chiba 278-8510, Japan

*Corresponding authors: E-mail, shinaoki@rs.tus.ac.jp

Contents

Table S1. Crystal data and structure refinement for **1** and **12**…………………………...**S3**

Figure S1. The results of MTT assays of A549, HeLa S3, U937, MOLT-4 and IMR-90 cells after the incubation with BAPTA and BAPTA-AM……………………………………………………………………………………**S5**

Figure S2. The results of UV/Vis absorption titrations of **1** with Zn^{2+}, Ni^{2+}, Co^{2+}, Cu^{2+}, Fe^{2+} and Al^{3+}………………………………………………………………………………………**S6**

Figure S3. The results of MTT assays of Jurkat cells treated with **1** and BAPTA for 24 h in the presence of Ni^{2+}, Co^{2+}, Fe^{2+}, Zn^{2+}, and Cu^{2+}………………………………………………**S7**

Figure S4. The results of MTT assays of Jurkat cells treated with BAPTA-AM in the presence of Ni^{2+}, Co^{2+}, Fe^{2+}, Zn^{2+}, and Cu^{2+}……………………………………………………… **S8**

Figure S5. The results of UV/Vis absorption titrations of BAPTA with Zn^{2+}, Ni^{2+}, Co^{2+}, and Cu^{2+} ……………………………………………………………………………………… **S9**

Figure S6. Effect of Z-VAD-fmk on the cell death in Jurkat cells induced by BAPTA-AM …………………………………………………………………………………...…**S10**

Figure S7. Structures of Mito-FerroGreen, FerroFarRed, and zinquin. …………………**S10**

Figure S8. Staining of Jurkat cells with zinquin to detect intracellular Zn^{2+} ions. ………**S11**

Table S1. Crystal data and structure refinement for **1** and **12**.

Compound No.	**1**	**12**
CCDC Deposition Number	2062877	2062878
Empirical formula	$C_{78}H_{60}N_{12}O_6$	$C_{82}H_{66}Cl_4N_{14}Ni_2O_{22}$
Formula weight	1261.38	1858.70
Temperature	123 K	123 K
Crystal system	Triclinic	monoclinic
Space group	P–1	P21/n
Unit cell dimensions	a = 9.925(5) Å	a = 23.755(3) Å
	b = 17.269(8) Å	b = 14.3713(18) Å
	c = 20.8607(10) Å	c = 24.465(3) Å
	α = 82.865(16)	α = 90
	β = 88.492(17)	β = 112.091(2)
	γ = 88.011(16)	γ = 90
Volume	3545(2) Å^3	7739.0(17) Å^3
Z	2	4
Density (calcd.)	1.182 g cm^{-3}	1.595 g cm^{-3}
Absorption coefficient	0.077 cm^{-1}	0.715 cm^{-1}
F(000)	1320	3824
Crystal size	0.300×0.150×0.020 mm^3	0.150 × 0.130×0.080 mm^3
Theta range for data collection	3.0 to 27.5°	3.0 to 27.5°
Index ranges	-12<=h<=12 -22<=k<=21 -26<=l<=24	-30<=h<=30 -18<=k<=16 -31<=l<=31
Reflections collected	28933	62388
Independent reflections	15530 (Rint = 0.0865)	17696 (Rint = 0.0490)
Completeness to theta = 27.477°	95.60%	99.70%
Absorption correction	multi-scan	multi-scan
Refinement method	Full-matrix least-square on F^2	Full-matrix least-square on F^2
Goodness-of-fit on F2	0.954	1.059
Final R indices [I>2sigma(I)]	R_1 = 0.1148<br>wR_2 = 0.2937	R_1 = 0.0646<br>wR_2 = 0.1521
R indices (all data)	R_1 = 0.2435<br>wR_2 = 0.3964	R_1 = 0.0906<br>wR2 = 0.1704

The comments on B-alerts of the crystal structures of **1** and **12** are summarized below;

(For the crystal structure of **1**, CCDC: 2062877)

PLAT026_ALERT_3_B
Author response: The crystal was low diffracting, although the best crystal was chosen.

PLAT084_ALERT_3_B
Author response: This is due to instability of the crystal, although best crystal was used in more than 10 attempts.
PLAT230_ALERT_2_B
Author response: The anomalous data for Hirshfeld test is an artefact in the structure. According to the other characterizations, there is no doubt that the structure of **1** has three 2,2’-bipyridyl units.
PLAT910_ALERT_3_B
Author response: This is likely due to beam-stop and this happens quite often when Mo radiation and the geometry of our goniometer are used.

(For the crystal structure of **12**, CCDC: 2062878)
PLAT910_ALERT_3_B
Author response: This is likely due to beam-stop. and this happens quite often when Mo radiation and our goniometer are used.

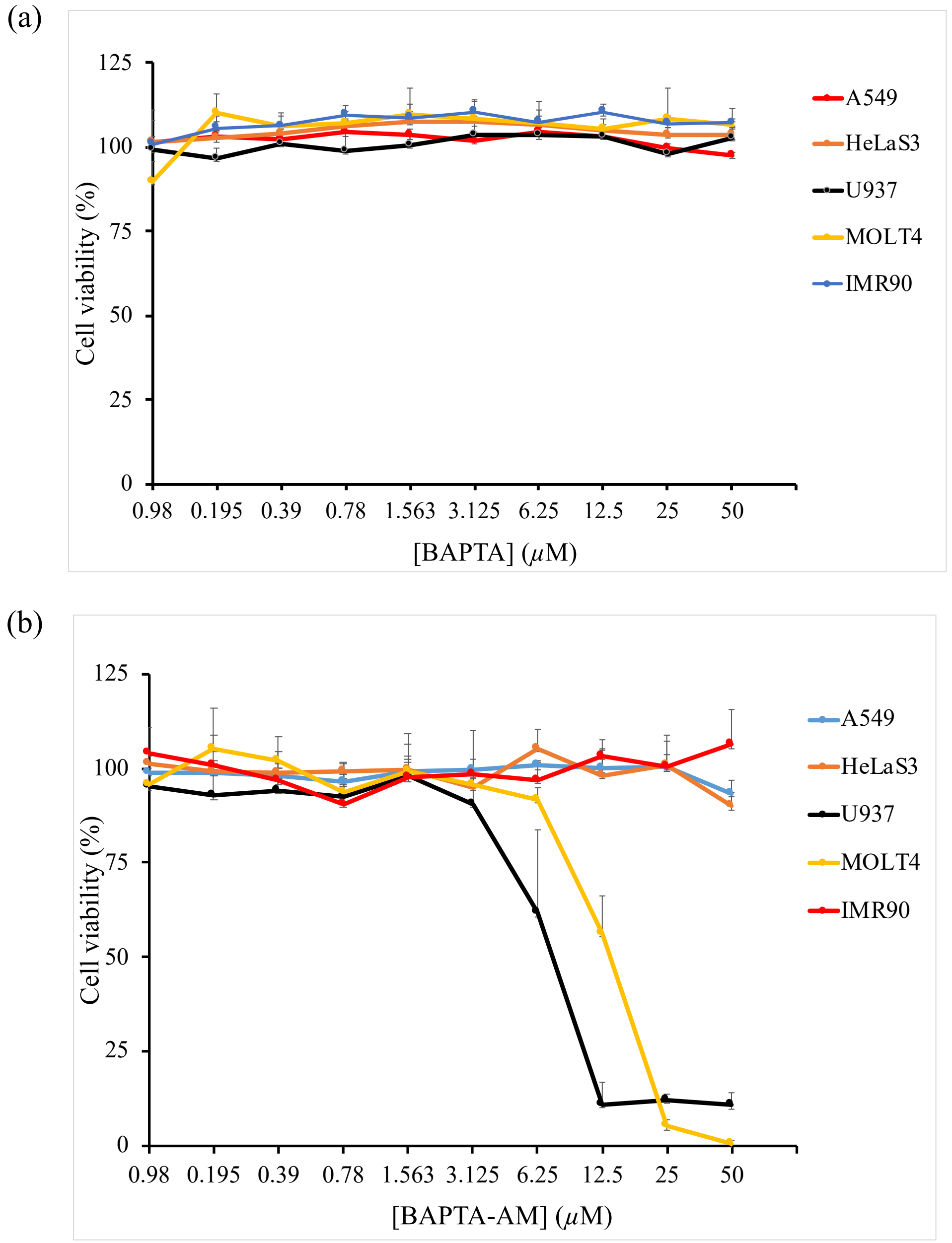

Figure S1. The results of MTT assays of A549, HeLa S3, U937, MOLT-4 and IMR-90 cells after the incubation with BAPTA (a) and BAPTA-AM (b) for 24 h.

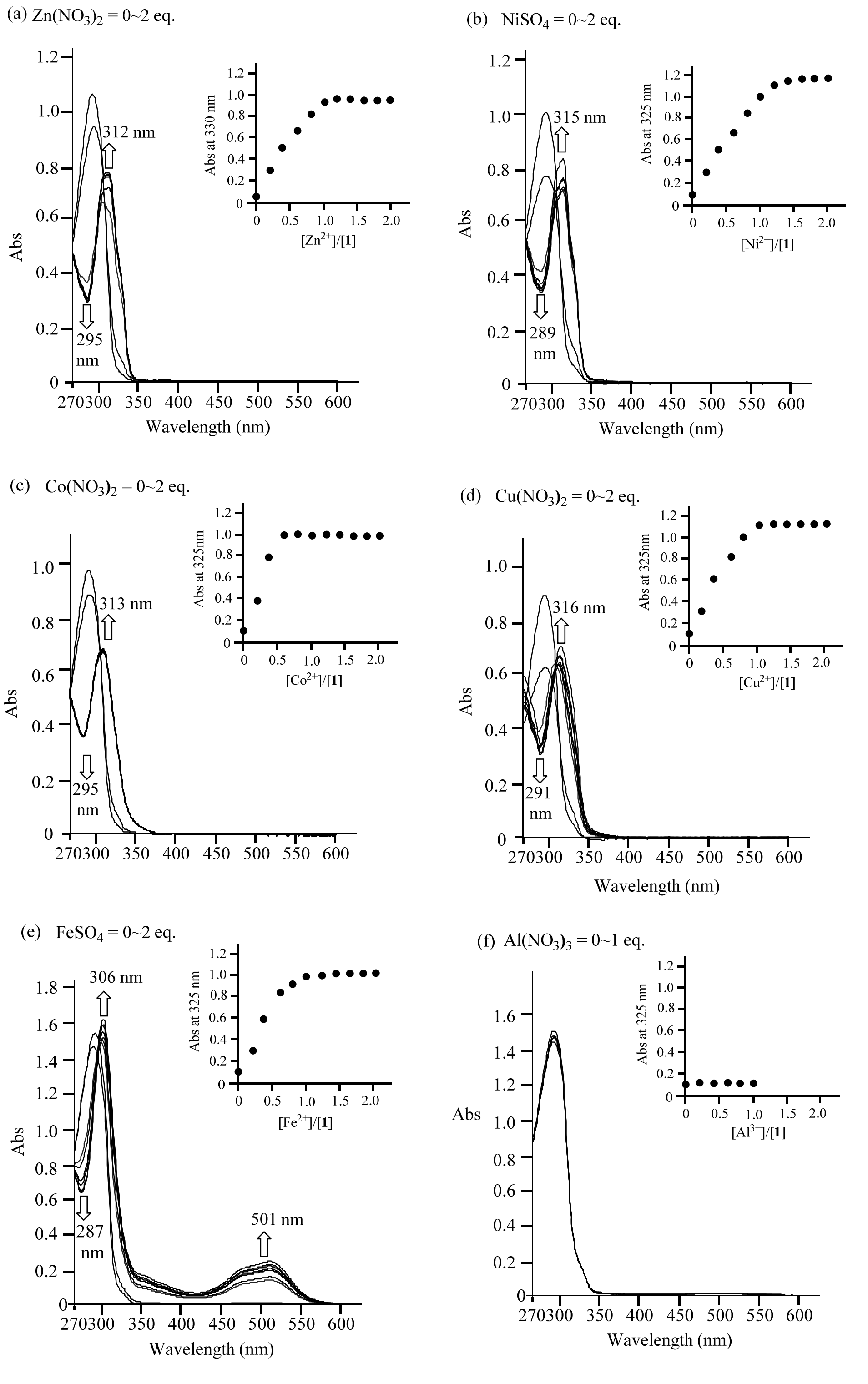

Figure S2. The results of UV/Vis absorption titrations of **1** (30 μM) with (a) Zn^{2+}, (b) Ni^{2+}, (c) Co^{2+}, (d) Cu^{2+}, (e) Fe^{2+} and (f) Al^{3+} in DMSO/30 mM HEPES (pH 7.4 with I = 0.1 ($NaNO_3$)) (7/3) at 37°C. Inset: The increase in Abs at given wavelength upon the addition of metal ions.

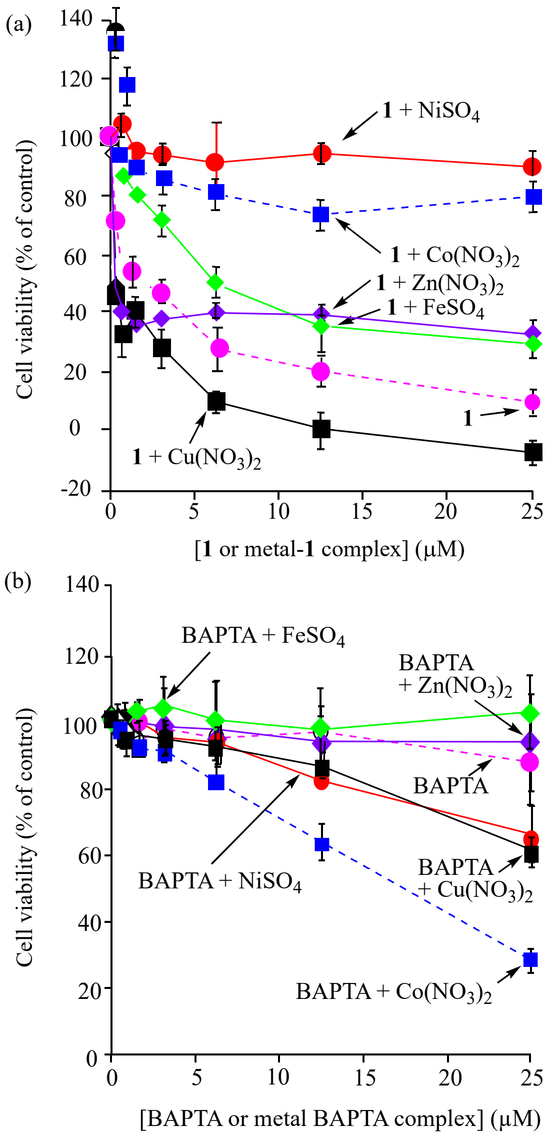

Figure S3. (a) The results of MTT assay of Jurkat cells treated with **1** alone (pink dashed curve with pink circles), in the presence of $NiSO_4$ (red plain curve with red circles), $Co(NO_3)_2$ (blue dashed curve with blue squares), $FeSO_4$ (green plain curve with green diamonds), $Zn(NO_3)_2$ (purple plain curve with purple diamonds) and $Cu(NO_3)_2$ (black plain curve with black filled squares), respectively, for 24 h ([**1**]: [M^{2+}] = 1: 1.5). (b) The results of MTT assay of Jurkat cells treated with BAPTA alone (pink dashed curve with pink circles), in the presence of $NiSO_4$ (red plain curve with red circles), $Co(NO_3)_2$ (blue dashed curve with blue squares), $FeSO_4$ (green plain curve with green diamonds), $Zn(NO_3)_2$ (purple plain curve with purple diamonds) and $Cu(NO_3)_2$ (black plain curve with black diamonds), respectively, for 24 h ([**1**]: [M^{2+}] = 1: 1.5) ([**1** or BAPTA]= 0.78-25 μM and [M^{2+}] = 1.17–37.5 μM).

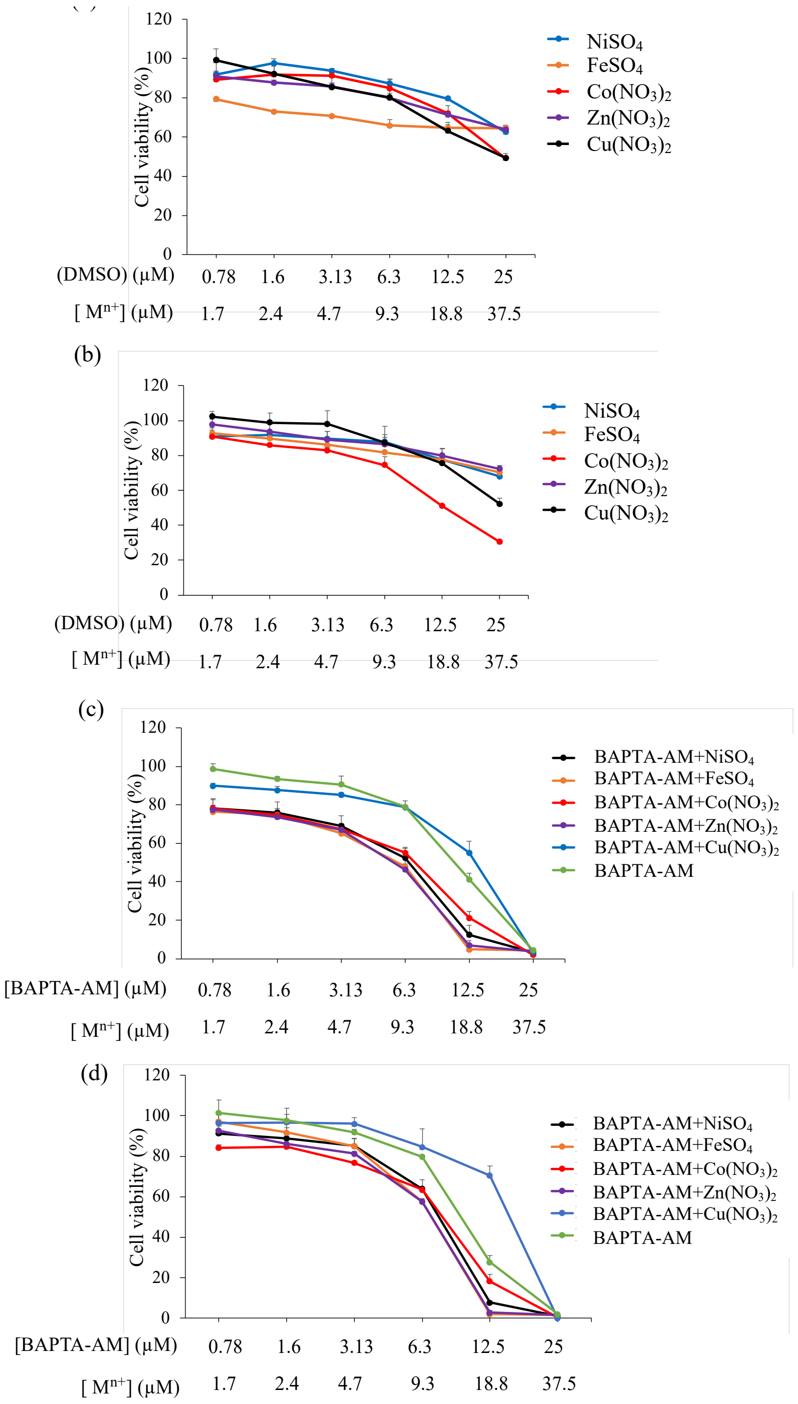

Figure S4. The results of MTT assays of Jurkat cells treated with DMSO alone and in the presence of $NiSO_4$, $Co(NO_3)_2$, $FeSO_4$, $Zn(NO_3)_2$ and $Cu(NO_3)_2$: $[M^{2+}]$ = 1: 1.5) after incubation for 24 h (a) and 48 h (b). The results of MTT assays of Jurkat cells treated with BAPTA-AM alone and in the presence of $NiSO_4$, $Co(NO_3)_2$, $FeSO_4$, $Zn(NO_3)_2$ and $Cu(NO_3)_2$ ([BAPTA-AM] : $[M^{2+}]$ = 1: 1.5) after incubation for 24 h (c) and 48 h (d).

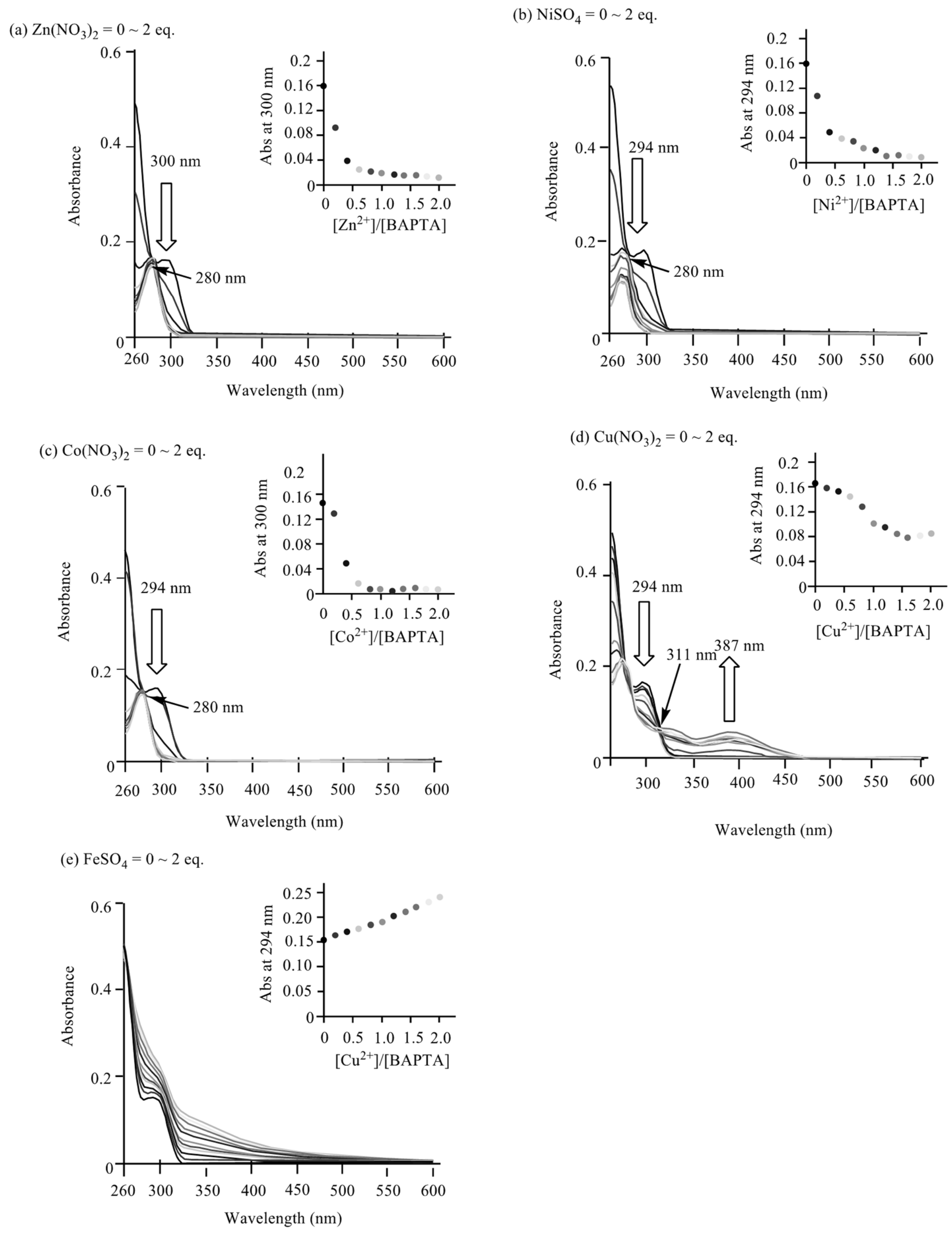

Figure S5. The results of UV/Vis absorption titrations of BAPTA (30 μM) with (a) Zn^{2+}, (b) Ni^{2+}, (c) Co^{2+}, (d) Cu^{2+} in DMSO/30 mM HEPES (pH 7.4 with $I = 0.1$ ($NaNO_3$)) (7/3) at 37°C. Inset: the increase in absorbance at given wavelength upon the addition of metal ions to BAPTA.

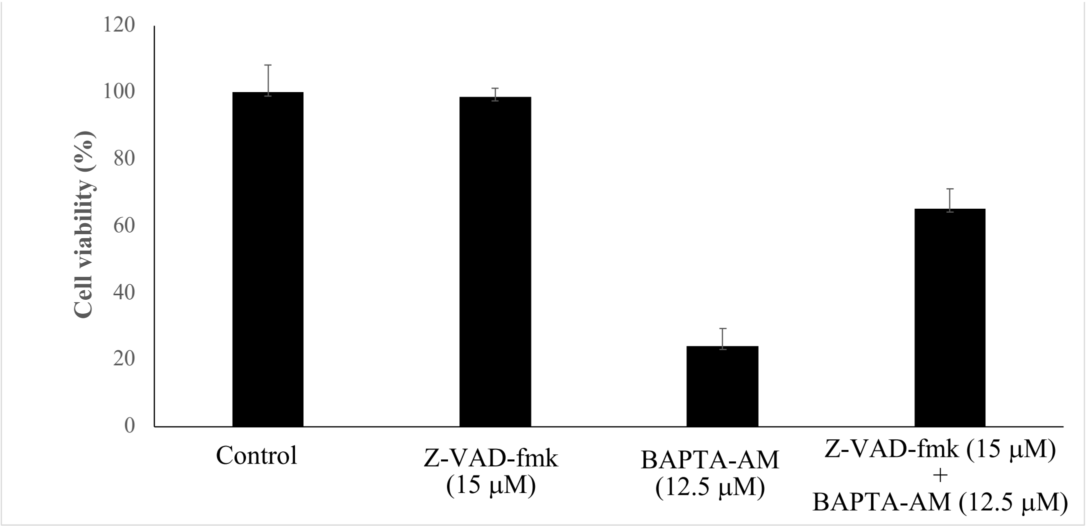

Figure S6. Effect of Z-VAD-fmk on the cell death in Jurkat cells induced by BAPTA-AM. Jurkat cells were incubated in RPMI containing BAPTA-AM (12.5 μM) for 24 h. Pre-treatment with Z-VAD-fmk at (15 μM) was conducted for 3 h prior to the incubation with BAPTA-AM.

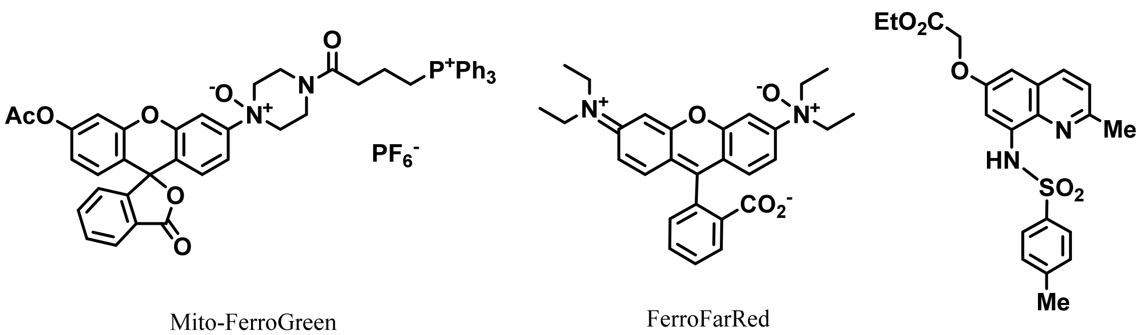

Mito-FerroGreen
(Ac-MtFluNox)
Em: 505 nm & Ex: 535 nm
after reaction with Fe^{2+}

FerroFarRed
(RhoNox-1)
Em: 646 nm & Ex: 662 nm
after reaction with Fe^{2+}

Zinquin
Em: 490 nm
Ex: 370 nm

Figure S7. Structures of Mito-FerroGreen, and FerroFarRed, and zinquin.

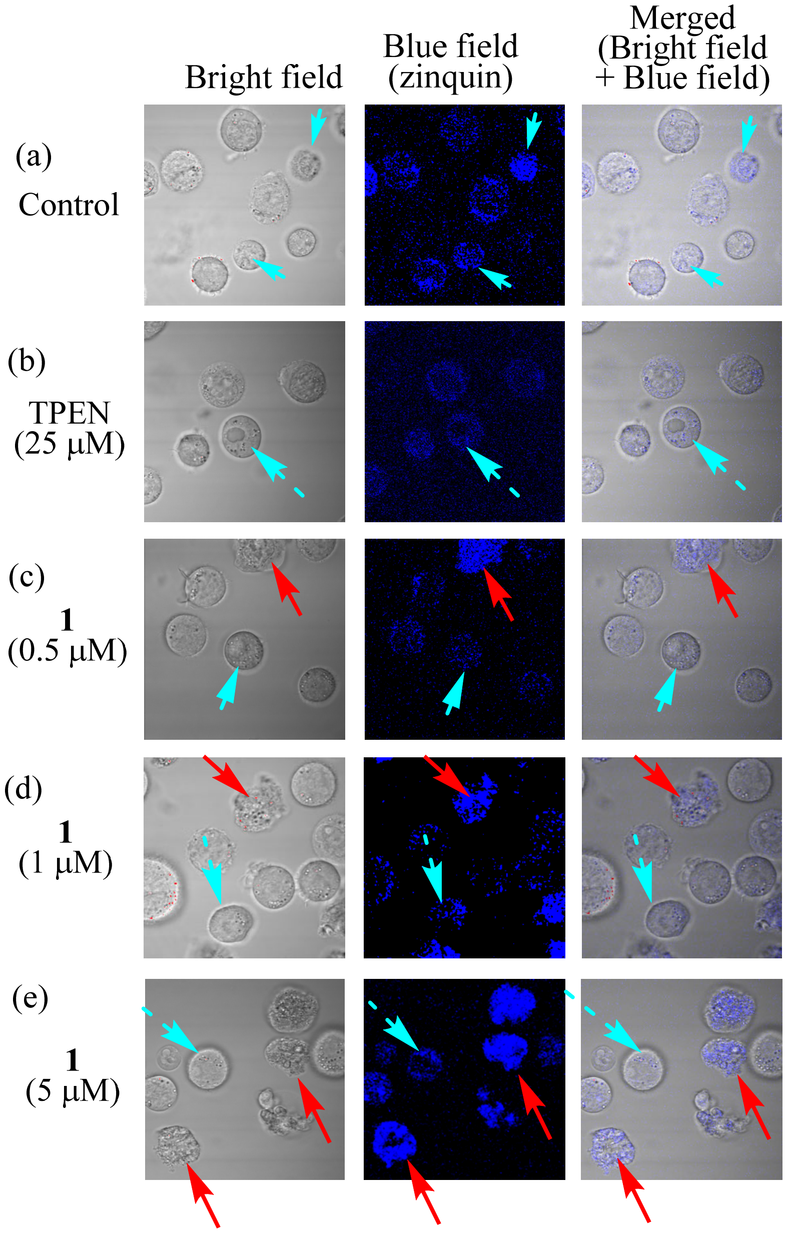

Figure S8. Staining of Jurkat cells with zinquin (25 μM) to detect intracellular Zn^{2+} ions. Jurkat cells were incubated with no ligand (control) (a), TPEN (25 μM) (b), **1** (0.5 μM) (c), **1** (1 μM) (d)**,** and **1** (5 μM) (e) for 24 h, prior to the incubation with zinquin (left: bright field image, middle; blue field image from zinquin, right: merged image). Red arrows indicate dead cells and light blue dashed arrows indicate live cells morphologically.
